# Real-time study of spatio-temporal dynamics (4D) of physiological activities in alive biological specimens with different FOVs and resolutions simultaneously

**DOI:** 10.1038/s41598-024-52152-x

**Published:** 2024-02-12

**Authors:** Aiswarya K. S., Sohela Sarkar, Smitha Vishnu, Rinsa S. R., Simran Negi, Nikhil Dev Narendradev, Rishica Harish Arora, Sreelakshmi Sanam, Anu P. V., Rahul Sharma, Satish Khurana, Jishy Varghese, Srinivasa Murty Srinivasula, Mayanglambam Suheshkumar Singh

**Affiliations:** 1 School of Physics (SoP), Indian Institute of Science Education and Research Thiruvananthapuram (IISER TVM), Thiruvananthapuram, Kerala 695551 India; 2 School of Biology (SoB), Indian Institute of Science Education and Research Thiruvananthapuram (IISER TVM), Thiruvananthapuram, Kerala 695551 India

**Keywords:** Light-sheet microscopy, Biological physics, Cell biology, Developmental biology, Haematopoietic stem cells

## Abstract

This article reports the development of a microscopy imaging system that gives feasibility for studying spatio-temporal dynamics of physiological activities of alive biological specimens (over entire volume not only for a particular section, i.e., in 4D). The imaging technology facilitates to obtain two image frames of a section of the larger specimen ($\sim \text {mm}$) with different FOVs at different resolutions or magnifications simultaneously in real-time (in addition to recovery of 3D (volume) information). Again, this imaging system addresses the longstanding challenges of housing multiple light sources (6 at the maximum till date) in microscopy (in general) and light sheet fluorescence microscopy (LSFM) (in particular), by using a tuneable pulsed laser source (with an operating wavelength in the range $\sim 420$–670 nm) in contrast to the conventional CW laser source being adopted for inducing photo-excitation of tagged fluorophores. In the present study, we employ four wavelengths ($\sim$ 488 nm, 585 nm, 590 nm, and 594 nm). Our study also demonstrates quantitative characterization of spatio-temporal dynamics (velocity—both amplitude and direction) of organelles (mitochondria) and their mutual correlationships. Mitochondria close to the nucleus (or in clustered cells) are observed to possess a lower degree of freedom in comparison to that at the cellular periphery (or isolated cells). In addition, the study demonstrates real-time observation and recording of the development and growth of all tracheal branches during the entire period ($\sim 95$ min) of embryonic development (*Drosophila*). The experimental results—with experiments being conducted in various and diversified biological specimens (*Drosophila melanogaster*, mouse embryo, and HeLa cells)—demonstrate that the study is of great scientific impact both from the aspects of technology and biological sciences.

## Introduction

Studies of embryonic development and cellular dynamics are of primary importance not only in the fundamental understanding of biological science but also in pharmaceutical and regenerative medicine. The study of the development of the *Drosophila* tracheal system has great implications in advancing our understanding of the biology behind the development of human vascular and respiratory systems which are known to be analogous with the fruit-fly trachea^1–3^. Several questions related to vascular and lung pathologies can be understood and thus, addressed by modeling and characterizing the underlying bio-molecular mechanisms that regulate the tracheal morphogenesis and its branching^1,4,5^. In insects, like (fruit-fly) *Drosophila melanogaster*, the circulatory system is open-type. To ensure quick and efficient delivery of oxygen to the entire parts or tissues of the body, the fruit fly establishes in place a unique network of progressively branching and interconnected tubes (known as the tracheal system)^6,7^. Shortly, the physiological activities of the tissues in every part and sub-part of the organism (both innermost and peripheral regions) are controlled and regulated by the tracheal system. Supplementary 1 gives the details of the tracheal system and its development. On the other hand, in cells, organelles like mitochondria undertake crucial physiological activities that include energy production, cell respiration, metabolic regulation, cell signaling, heat generation, and mediate in cell growth and death^8,9^. Mitochondria are highly dynamic and motile organelles that undergo frequent events of fusion and fission^9–11^. Understanding the dynamics of this organelle provides vital insights into both physiology and patho-physiology including metabolic diseases^10^. Also, locating different organelles in a single sample and studying how they work together to attain different functions has great importance in pathophysiology. Meanwhile, research studies in stem cells—that are the special human cells that can develop into many different cell types (ranging from muscle to brain cells)^12^—remain one of the promising research fields in regenerative medicine. Understanding the localization of Hematopoietic Stem Cells (HSCs), which are the stem cells that have the capacity to self-renew and differentiate into any of the blood cell lineages of the adult hematopoietic hierarchy (called hematopoiesis)^13^, are crucial in further characterizing the niche that supports and maintains HSC development. In mice (*Mus musculus*), these cells are first detected as hematopoietic cells (HCs) in the endothelial wall of the dorsal aorta within the aorta-gonad-mesonephros (AGM) region starting at embryonic day 10.5 (E10.5).

State-of-the-art imaging technologies adopted for developmental biology and its studies are limited only to fluorescence imaging and confocal microscopy^14–16^. This is true for the case of cellular and molecular biology as well. Both of the technologies suffer from serious drawbacks. Fluorescence imaging gives faster (or real-time) imaging and higher penetration/imaging depth but the spatial resolution obtainable is limited. On the other hand, confocal microscopy—which is fundamentally based on raster (point-by-point) scanning of a given specimen by a sharply focusing optical spot—remains the gold-standard imaging technology giving the best possible spatial resolution for biological studies^17^. Because of the raster scanning nature, confocal microscopy imaging technology fails to give images in real-time. Shortly, the achievable frame rate is relatively low ($\sim$mins.), whereby, the achievable frame rate is primarily determined by the pre-specified physical dimension of interest of the specimen (for imaging), i.e., field of view (FOV)^18,19^. Again, the dynamics of biological processes are of diversified range from nano- to milli-scale (both in space and time). From such technological limitation, it is not possible to observe the tracheal development at the various segments at a given instant of time and thus, fails to establish the spatio-temporal correlation of tracheal developments in various segments of an embryo. One may say that the prevalent advances in understanding of developmental biology can be established only from observations of the biological activities in the corresponding segments at different instants of time. In addition, from technological aspects and its limitations (in confocal microscopy), the imaging specimens are mandated to be cut into thin slices and subsequently, to be fixed. This gives an inherent limitation that restricts conducting the experiments in alive biological specimens or alive biological conditions. Conclusively, one may say that the present advances in understanding biology and its complexity are derived only from the studies in fixed (or not alive) biological specimens, i.e., understanding of physiological activities in alive is concluded from studies being conducted not in alive conditions, that may be erroneous certainly to an extent. There are several other serious drawbacks including photo-toxicity and photo-bleaching that greatly affect the specimen (even damage). To conclude, advancement in technology development of pre-eminent imaging modality—with an achievable sub-microscopic spatial resolution at higher penetration depth ($\sim$ mm) in real-time that holds promises to track minute changes from different parts/sections (at diversified scales or resolutions simultaneously) of the imaging specimen in intact (not in fixed) condition—becomes utmost scientific importance. In this direction, light sheet fluorescence microscopy (LSFM)—an emerging non-destructive microscopy imaging technology to generate multi-dimensional (sub-microscopic resolution) images of fully intact biological specimens (size $\sim$ mm) in real-time—stands as a promising imaging tool^20–26^.

Recently, studies were reported that demonstrated real-time imaging of different stages of development of the tracheal system as well as mitochondria movement using LSFM imaging modality^27,28^. However, in the reported studies, the adopted LSFM imaging technologies enable to give a single image frame at a given instant of time. Consequently, the reported studies fail to observe the spatio-temporal correlationship of detailed tracheal system development in the various sections and/or sub-sections of the entire embryo. For details, see Supplementary 1. Similar is the case for mitochondria and their movements within the cells. Even though LSFM successfully addresses the persisting technological challenge of imaging alive specimens in real-time, the existing LSFM technologies are limited to adapt a limited number of optical sources ($\sim 6$ at the maximum, to the best of our knowledge) that is resulted from limited physical space available within the imaging system^29^. With this limited number of optical wavelengths to induce photo-excitation of the tagged fluorophores, only selective physiological activities of the specimen can be studied^18,19^. In this report, we present a unique technology that adapts a tuneable pulse laser source (pulse width $\sim 6$ ns), instead of conventional CW-laser sources, which permits to select any optical excitation wavelengths in a wide range $\sim 420-670$ nm. In addition, our imaging technology, which we name as multiple wavelength simultaneous multiple-level magnification selective plane illumination microscopy (M$\lambda$-sMx-SPIM), enables us to provide two individual image frames of a single slice of an alive biological specimen (not in fix) at different magnification levels, different spatial resolutions, and different FOVs simultaneously at a given instant of time that remains as a technological challenge (see Supplementary 2, for details)^30^. Recently, we reported dual-arm LSFM imaging modality but the studies were not only limited to fixed (not alive) samples but also employed a single optical wavelength^18,19^. Shortly, our proposed M$\lambda$-sMx-SPIM differs from the existing LSFM imaging techniques primarily in two different aspects: (i) to provide photo-excitation of tagged fluorophores of any (optical) excitation wavelengths in a wide range $\sim 420-670$ nm in contrast to a specific and limited number of wavelengths ($\sim 6$ optical wavelengths), (ii) to provide two individual image frames of a single slice of biological specimen at different magnification levels, different spatial resolutions, and different FOVs simultaneously.

Experimental validation studies were conducted in various and diversified biological specimens, namely, *Drosophila melanogaster*, mouse embryo, and HeLa cells. The experimental results demonstrate that one can observe and record real-time spatio-temporal dynamics of (diversified) biological activities— say, development of the tracheal system (in *Drosophila melanogaster*) and organelles activities (localization and measurement of velocity of mitochondria)—uniquely achievable at different fields of view (FOVs) and different magnification levels (or different spatial resolutions) simultaneously, i.e., at the same instant of time. With our M$\lambda$-sMx-SPIM, one can observe simultaneously the development of the tracheal system over the entire body (*Drosophila* embryo, at larger FOV (but at lower magnification and resolution) and in the various sub-sections/segments of the body (at lower FOV but with higher magnification and higher resolution). This unprecedentedly enables one to observe and study the spatio-temporal correlation in the development of the tracheal system in various segments of the embryos (over the entire time interval of the developmental process that lasts a few hours). Again, our M$\lambda$-sMx-SPIM enables us to visualize the endothelial cells and hematopoietic cells in a fixed mouse embryo which confirms the localization of HCs in the embryo, as well as to image different organelle, namely, microtubule and nuclei in HeLa cell line. For the case of mitochondrial dynamics, our home-built imaging system enables us not only to observe and record the fast dynamics (velocity) of cellular organelles (mitochondria) but also to capture images of the entire (specific) cells of interest and the cellular dynamics (simultaneously). Studies on this subject of great scientific importance have not yet been reported to date which is solely due to the lack of technological advances (more particularly, in imaging).

## Results

### Tracheal branching in *Drosophila melanogaster*

**Figure 1:**
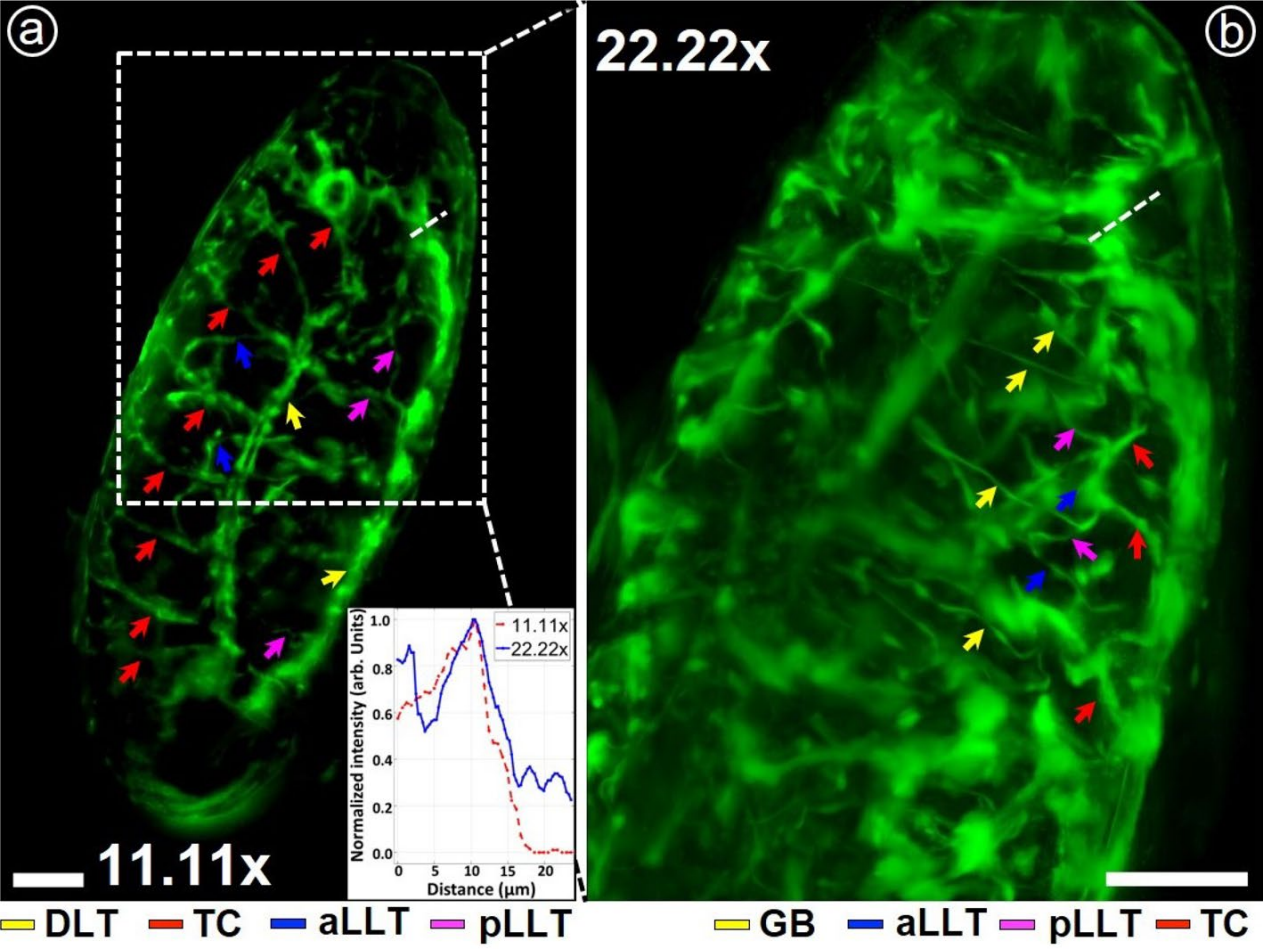
The projection images at $11.11\times$ and $22.22\times$ magnification on XY plane. Some of the observed branches are pointed by arrowheads of different colours for different types of branches. DLT: Dorsal longitudinal trunk; TC: Transverse connective; aLLT: Anterior lateral longitudinal branch; pLLT: Posterior lateral longitudinal branch; TC: Transverse connective; GB: Ventral ganglionic branch. Line-plot—showing the variation of normalized intensity along a randomly chosen line from the same region of interest in both magnifications (as they marked in the figures)—is included as an inset. Scale bar: 50 $\mu m$.

**Figure 2:**
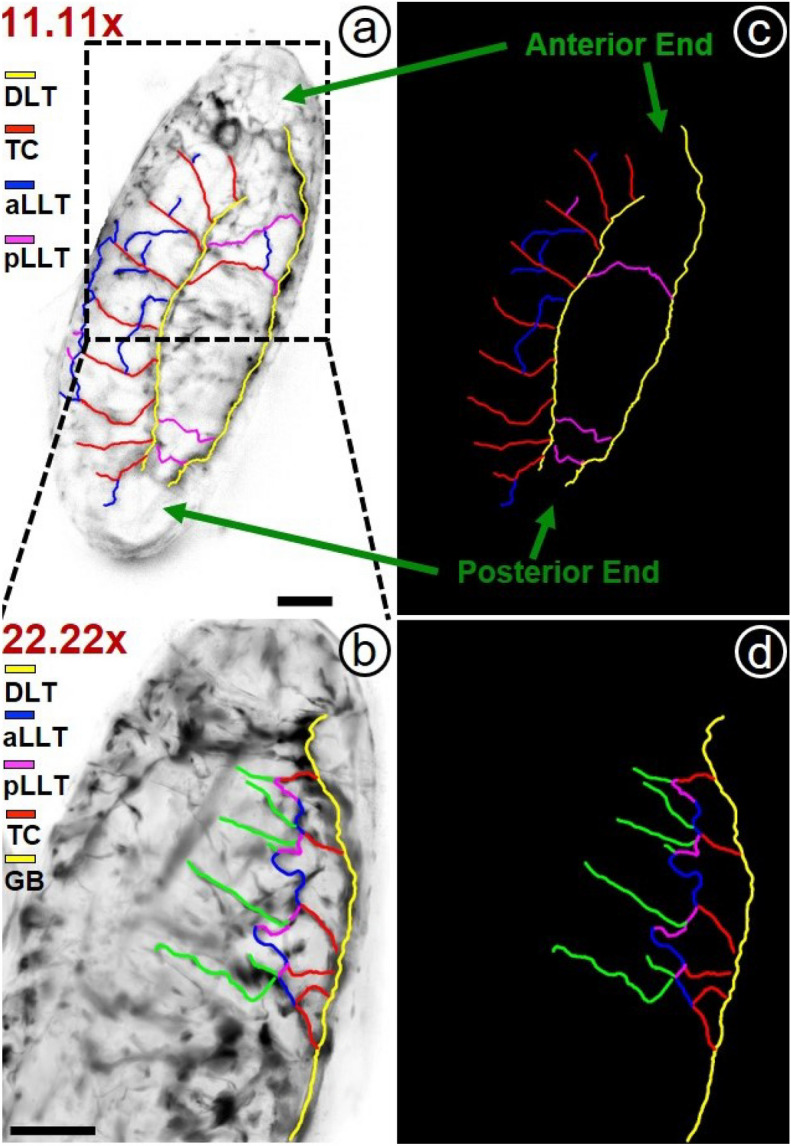
Mapping of tracheal branches in Stage 15 of embryonic development of *Drosophila melanogaster* (fixed sample) at two different magnifications ($11.11\times$ ((**a**) and (**c**)) and $22.22\times$ ((**b**) and (**d**)). Figures depict the tracing of tracheal systems that are observed at two different magnifications or resolutions ((**c**) and (**d**)). Scale bar: 50 $\mu m$.

**Figure 3:**
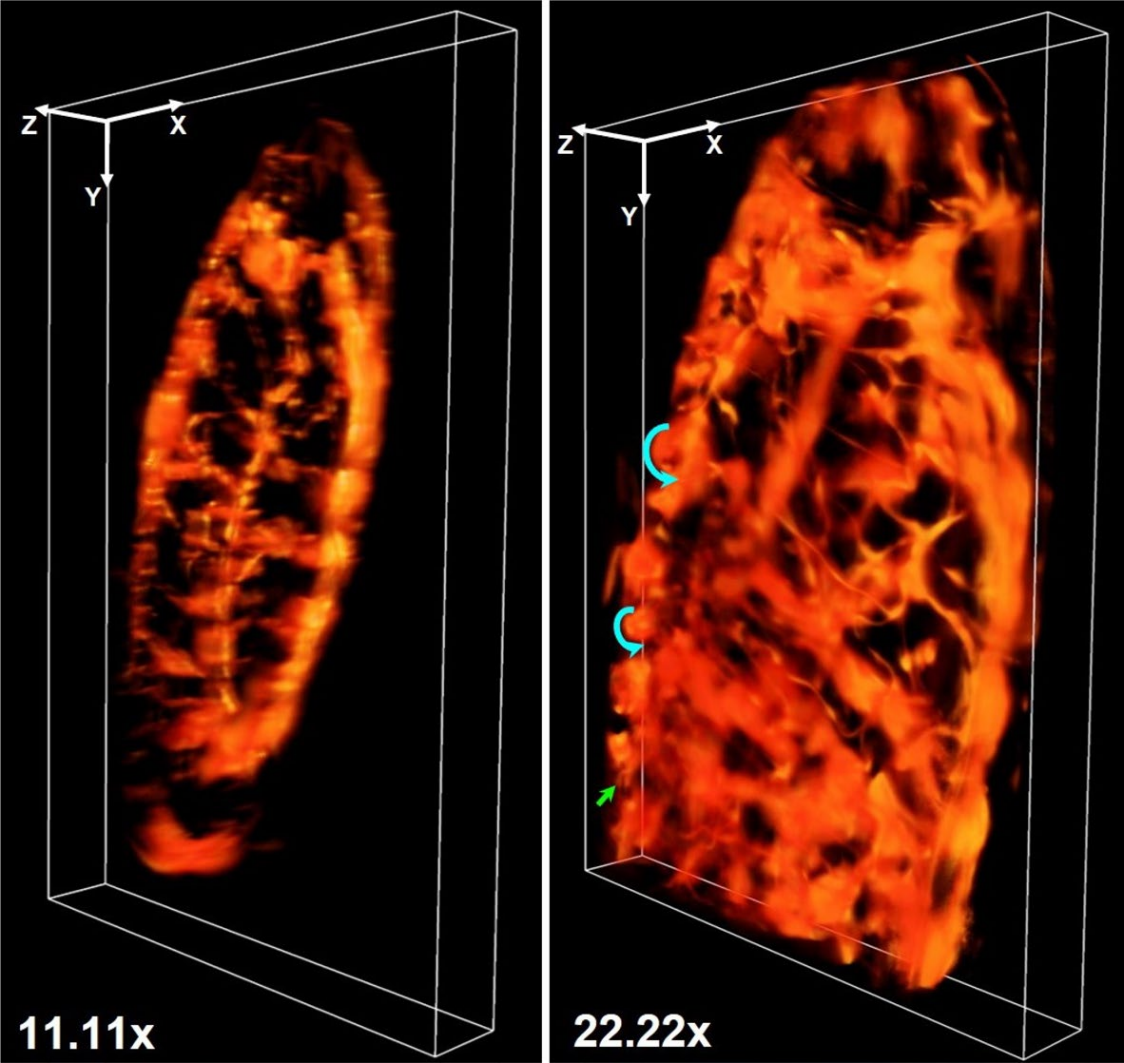
3D reconstructed images of the tracheal system of *Drosophila melanogaster* (at Stage 15). The arrows indicate the dorsal branches spread in the volume.

Figure 1 presents the experimental results for experiments being conducted in a fixed sample (more specifically, *Drosophila* embryos at Stage 15 of embryonic development). Figure 1a and b give projected images (in *xy*-plane) corresponding to magnifications obtained at $11.11\times$ (detection Arm-I) and $22.22\times$ (detection Arm-II) respectively. For this magnified view, the detection arm (Arm-II) was kept fixed over the area as indicated by dashed lines shown in Fig. 1a). These projected images were generated from a sequence of 2D images covering the entire volume of the specimen while each stack of the 2D images was undertaken a sequence of image processing for improving the obtainable image quality (before taking the projection). The details with the associated results are provided separately in Supplementary 5. In the figure (Fig. 1), one observes the structures of the specimens with better clarity and higher resolution in higher magnification image that are clearly observable from the line-plot included as an inset in the figure (Fig. 1a). The line plot from the same region of interest from both magnifications shows that the $22.22\times$ magnification contains more information, especially from the narrow tracheal branches, than the $11.11\times$ magnification. There is a slight difference in the two images as they are corresponding to two different sides of the specimens that are not perfectly symmetric. The different branches (which are marked by different colour arrowheads) can be seen very clearly in Fig. 1 but for a better understanding of the tracheal branches they have been traced using the NeuronJ plugin in ImageJ. Hence in the figures (Fig. 2a and b), we depict the tracing of tracheal branches being overlaid on the projected images of the specimen. Figure 2c and d give the tracheal branching without overlying in the background. It is observed that the image obtained at magnification $\sim 11.11\times$ can depict the entire embryo (but at a lower resolution) and we can discern the different transverse connectives of the tracheal branches from the dorsal longitudinal trunk, namely, DLT, TC, aLLT, and pLLT. The image at $22.22\times$ magnification gives better resolution (but at lower FOV). With this higher magnification, one can distinctly observe the fine branches or structures. Figure 3 presents the corresponding 3D views of the sample obtained at two separate magnifications ($11.11\times$ and $22.22\times$) simultaneously at an instant of time. The arrowheads in Fig. 3 show the branches spread in the volume. The $360^\circ$ panoramic views of the 3D reconstructed images at both magnifications are given separately in Supplementary 6. These experimental results demonstrate the capability of our imaging system for imaging the entire volume of a given specimen at two separate fields of view (FOVs) and resolutions simultaneously or at one instant of time.

**Figure 4:**
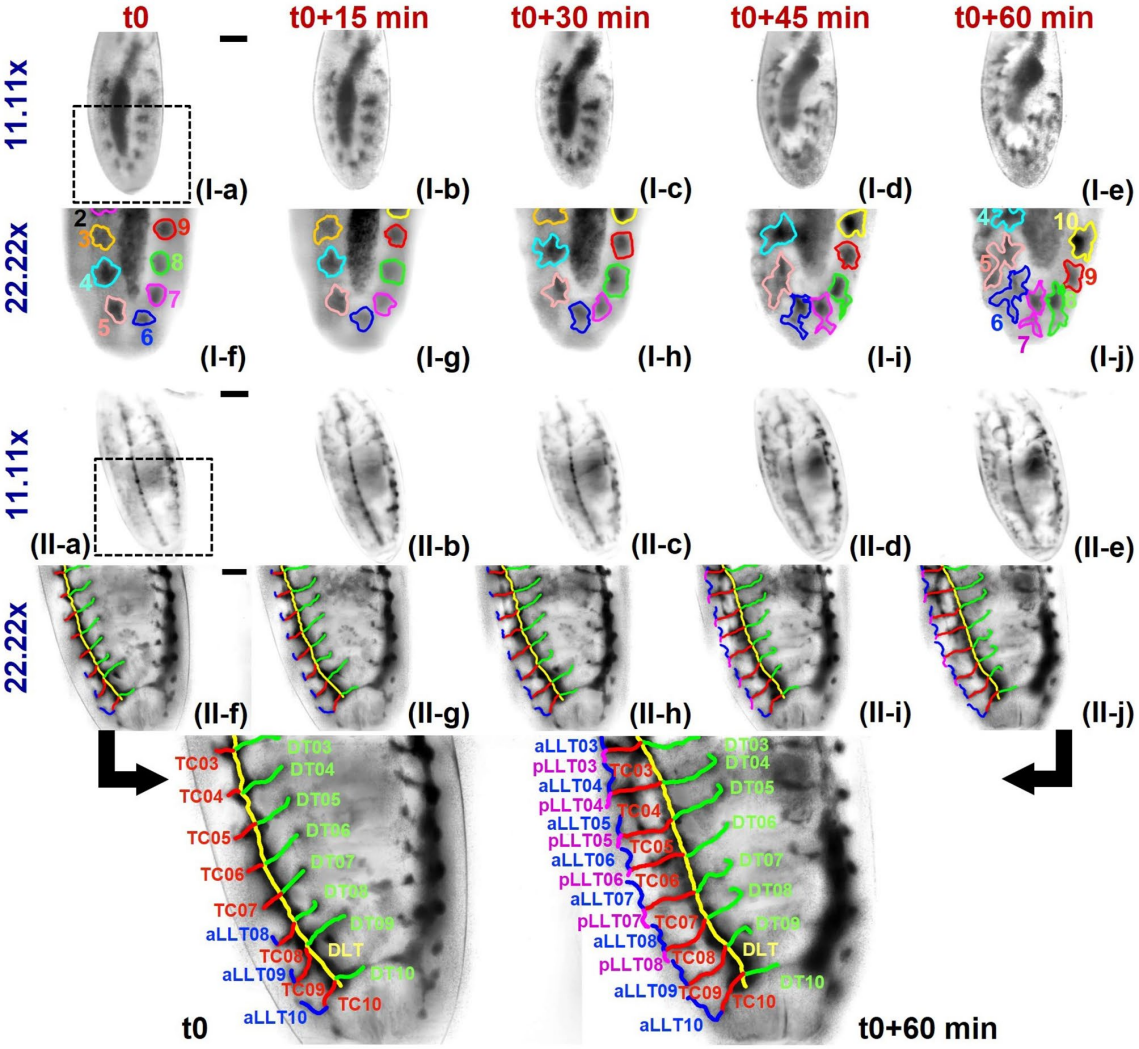
Tracing of tracheal branching of embryonic development for Sample I: Stage 11 to Stage 12 (I(**a**–**j**)) and Sample II: Showing progression from late Stage 13 to Stage 15 (II(**a**–**j**)). The magnified images of Sample II at *t*0 and $t0+60$ min shows the different branches with labels where DLT: Dorsal Longitudinal trunk, DT: Dorsal tracheal branch, TC: Transverse connective, aLLT: Anterior lateral longitudinal branch, pLLT: Posterior lateral longitudinal branch. Scale bar: 50 $\mu m$.

**Figure 5:**
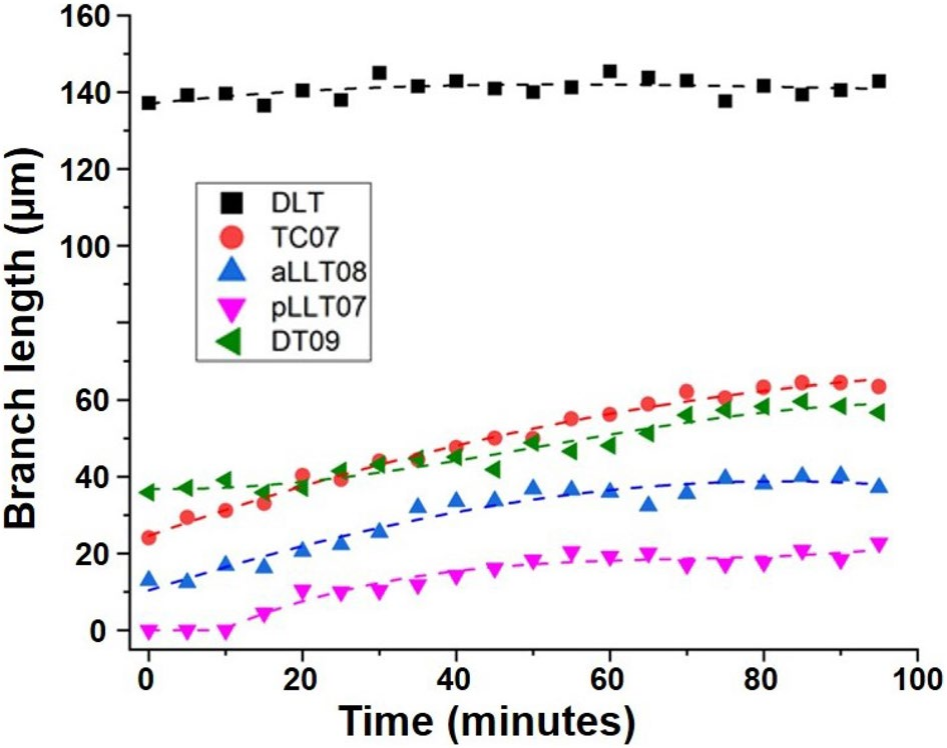
Variation of the lengths of various tracheal branches with time for *Drosophila melanogaster* (Sample II).

Figure 4 presents the experimental results with experiments being performed in alive biological specimens (*Drosophila melanogaster*). Experiments were conducted in separate groups of live *Drosophila* embryos (more specifically, at different stages of embryonic development). Figure 4(I(a–j)) present images of the sample at embryonic development Stages 11 to 12 (say, Sample I) while Fig. 4(II(a–j)) depict images corresponding to the sample progressing from late Stage 13 to Stage 15 (say, Sample II). The embryo’s activity was recorded at an interval of 5 min for an entire duration of 60 min (for Sample I) and 95 min (for Sample II). In Fig. 4, we present only certain selective images—that are recorded at the time interval of 15 min— while the complete information of images corresponding to recording at an interval of 5 min is given in Fig. 5. Figure 4(I(a–e)) presents images obtained at $11.11\times$ magnification while Fig. 4(I(f–j)) corresponds to $22.22\times$ magnification (Sample 2). Similarly, Fig. 4(II(a–e)) depict images obtained at $11.11\times$ magnification while Fig. 4(II(f–j)) correspond to $22.22\times$ magnification (Sample II). The corresponding time-resolved spatio-temporal dynamics (i.e., video) of the tracheal branching for both samples are given separately in Supplementary 7. Over the duration of one-hour imaging of tracheal development (in Sample II), it is observed that the *Drosophila* embryo develops from Stage 10 to 12 where we can see all of the ten tracheal pits (or placodes) are re-positioned inside the embryo. The tracheal pits that are invaginating also begin to elongate giving rise to a dorsal and a ventral stem. The stem towards the ventral side also shows a slight bifurcation giving rise to a small posterior branch and a small anterior branch growing into branches while re-arranging to cover the entire region of the whole embryo. This is in good agreement with reported studies (see Supplementary I). Here, the image at $11.11\times$ gives the complete view of the whole embryo where the ten tracheal pits are clearly observed to be re-positioned or re-arranged. At the same time, in the image with $22.22\times$ magnification that corresponds to the magnified view of the region marked by the square box indicated in Fig. 4(I-a), we observe each tracheal pit also developing the bifurcating stems. Similarly, live imaging of the development of the tracheal system from late Stage 13 to Stage 15 was conducted with $11.11\times$ and $22.22\times$ magnifications for a duration of 95 min (images at 15 min intervals for 1 h shown in Fig. 4(II(a–e)) and Fig. 4(II(f–j)) respectively). The lower magnification image covers the entire embryo (but at lower resolution) while higher magnification gives the higher resolved images of branch formations (but at the cost of FOV). In the time interval from *t*0 to $t0+95$ min, we observe the formation of some new tracheal branches as well as the continuation of growth and movements of the existing branches. It is observed that the photo-bleaching over the time of imaging is relatively low (See Supplementary 4). We characterize quantitatively the growth in length of the tracheal branches with respect to time.

For a better understanding of branch growth, the quantification of an increase in branch length over time is studied for (several) branches over certain regions of interest (for Sample II). The graphical plot of branch length vs. time is given in Fig. 5. In the figure, we can distinctly observe that the branch lengths are increasing and saturated, i.e., tracheal branches are growing with time and attaining maturities at different time intervals. The DLT branch length is almost saturated from the beginning. During embryonic development of Drosophila melanogaster, around late Stage 13 (early Stage 14), the individual portions of the dorsal trachea originating from the 10 tracheal placodes fuse completely to give rise to a single dorsal longitudinal trunk (DLT) of the tracheal system on each side of the body. Although minor cellular movements continue within the DLT, at late Stage 13 (where the imaging of Sample II begins), the DLT is a fully formed continuous tube. Therefore, we do not observe any further visible longitudinal growth in it. Meanwhile, during Stages 13-15, several new tracheal branches emerge from the DLT sequentially. The transverse connectives (TC) and the dorsal tracheal branches (DT) emerge simultaneously from the DLT towards the transverse and dorsal sides of the embryo respectively. Towards the later part of Stage 15, the anterior and posterior lateral longitudinal branches (aLLT and pLLT) extend to fuse with the lateral longitudinal branches of the adjacent placode, along with the emergence of tracheal terminal cells (TTC—not depicted in the images). The LLTs are smaller branches compared to both DTs and TCs. Sample II depicts an embryo between the Stages 13-15. Therefore, we observe the most noticeable and significant growth in the transverse connectives (e.g., TC07 branch), followed by the dorsal tracheal branches (e.g., DT09). We also observe the emergence of the lateral longitudinal branches (e.g., aLLT08 and pLLT07) and capture a portion of their growth dynamics within the time frame of our imaging session. In short, in the figure, we observe that TC07 increases significantly while the DLT shows almost no increase. A comparison of branch lengths of all the branches in Sample II is given separately in Supplementary 8 (see Supplementary Fig. S5-II). For the quantitative analysis of Sample I, the areas of the placodes (at the initial and final time of imaging) are calculated, and the comparison is given in Supplementary 8 (see Supplementary Fig. S5-I).

In a true sense, simultaneous imaging of a single frame/slice in complex three-dimensional biological samples such as the live Drosophila embryo is of great importance but remains as a technological challenge. The inclusion of a higher magnification objective in the setup suggests the potential to scrutinize developmental processes at the cellular level, deep within the tissues, with precise spatio-temporal kinetics. The ability to image the same 3-D sample simultaneously using an objective of lower magnification allows us to continuously monitor the overall status of the live sample. This can allow biologists to track the developmental progress, viability of the embryo, morphological (physical) changes at the surface of the embryo, etc. while tracking in parallel the corresponding cellular changes at the deeper layers inside the embryo. Our set-up, for simultaneous imaging of Drosophila embryos (and other 3-D live biological samples) at two different magnifications, therefore, is necessary for biologists during experimentations and is also advantageous, allowing parallel data collection of the cellular processes as well as the status of the experimental sample. This is to note that, even though we present images obtained at $11.11\times$ and $22.22\times$ (in the present study), objective lenses adapted in the two separate or individual detection arms (Arm I and Arm II) are not limited to the two magnifying powers. Instead, the objective lenses can be changed independently and thus, one can obtain images of specimens not only at any magnifications or resolutions but also with any possible combinations that can be achieved by adapting appropriate objective lenses of interest (and the combinations) in the detection arms.

### Dynamics of mitochondria in HeLa cells

**Figure 6:**
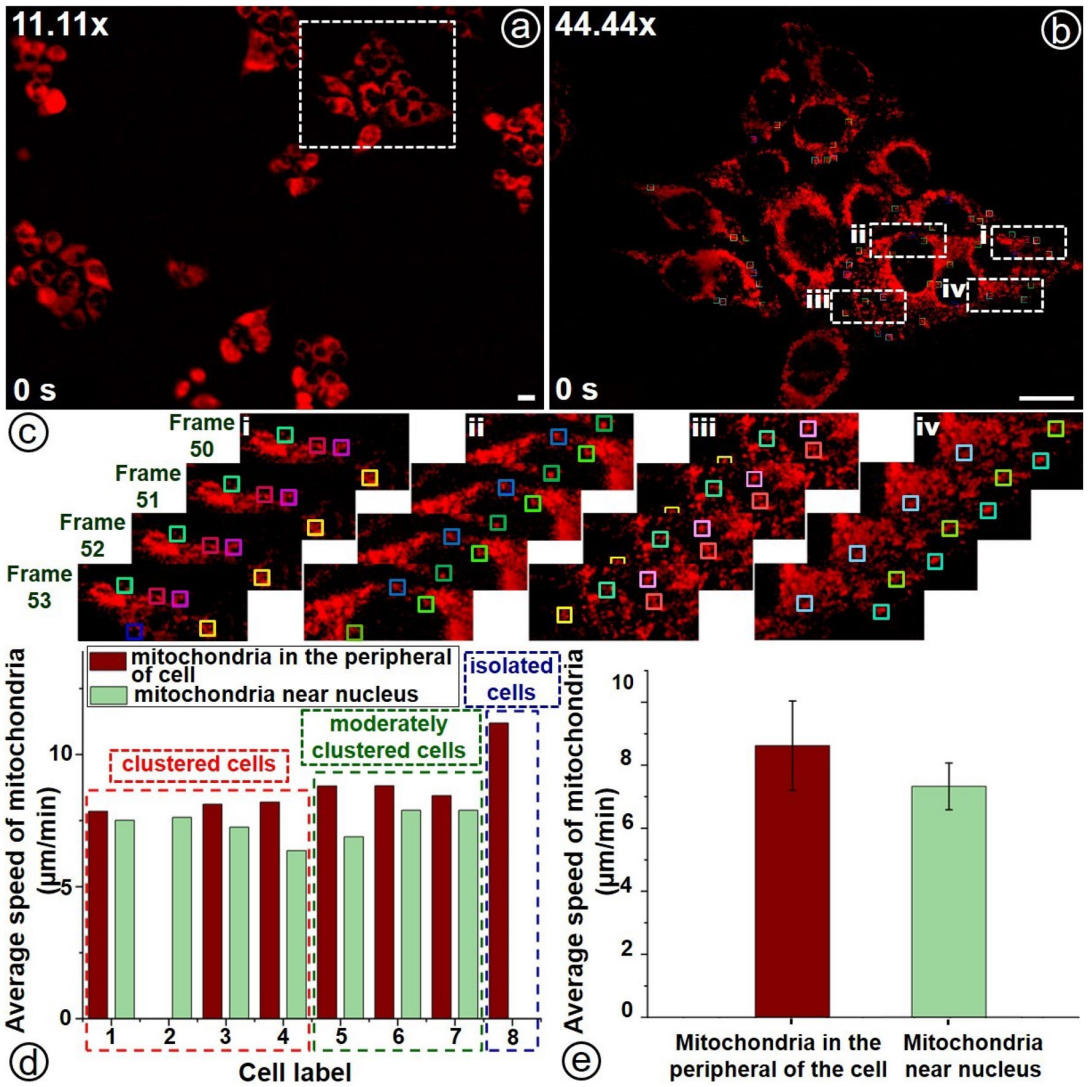
Images of mitochondria of HeLa cells that are obtained at $11.11\times$ (**a**) and $44.44\times$ (**b**) magnifications but simultaneously. (**c**) Zoomed-in views for detection of various mitochondrion (marked in coloured boxes) in each frame at four different regions. Comparison of (**d**) average speed ($\bar{v}$) of mitochondria in clustered cells, moderately clustered cells and isolated cells and (**e**) average speed ($\bar{v}$) of mitochondria at the periphery of the cell and near the nucleus. Scale bar: 20 $\mu m$.

To validate experimentally the feasibility of our M$\lambda$-sMx-SPIM to study spatio-temporal dynamics at the cellular level, we conducted experiments on HeLa cells as the imaging specimen, more specifically, to study the spatio-temporal dynamics of organelles (mitochondria). Figure 6 presents images obtained at the initial time ($t0=0$ s). Figure 6a and b present the corresponding images obtained at $11.11\times$ magnification (detection Arm I) and $44.44\times$ magnification (detection Arm II). The marked region (indicated in Fig 6a) shows the field of view corresponding to Fig. 6b ($44.44\times$ magnification). The lower magnification view enables us to locate selectively the desired cells from a cluster or large number of cells that is in agreement with the arguments presented in Supplementary 2. Imaging was performed for 98 seconds, and 222 frames were recorded (in the detection Arm II) while 863 frames were recorded in the detection Arm I. The lower magnification ($11.11\times$) fails to provide enough resolution to observe the movements of organelle (mitochondria). But, at higher magnification ($44.44\times$), one can clearly observe and detect the mitochondria and their spatio-temporal dynamics. For the quantification of the mitochondrial spatio-temporal dynamics, speeds of different mitochondria—as indicated by marked boxes of different colours (various regions are zoomed-in in Fig. 6c)— were calculated by using a custom-made MATLAB program. The underlying algorithm with flowchart is given in Supplementary 9 (see Supplementary Fig. S6). Video showing the time-resolved dynamics of mitochondria and its tracking is given separately in Supplementary 10. It is observed that the speed of mitochondria near the nucleus (distance from the nucleus $<2 \mu m$ ) and the periphery of the cell (distance from the nucleus $>2 \mu m$) were tabulated separately for different cells. Mean and standard deviation in the measurements of speed were calculated for correlation studies.

As it is clearly visible in Fig. 6b, some cells are observed to be clustered together while some are isolated. The mean speed of mitochondria—both near the nucleus and periphery of the cell—for each of the cells are plotted in Fig. 6d. The cells are marked and labeled separately as clustered, moderately clustered, and isolated. The labeling of the cells and the mitochondria are presented in Supplementary 11. The average speed ($\bar{v}$) of mitochondria in the clustered cells ($\sim 8.03 \pm 1.05$ $\mu m/min.$) is found to be comparatively lower than that of the moderately clustered cells ($\sim 8.68 \pm 0.64$ $\mu m/min.$). The speed is measured to be much higher for isolated cells ($\sim 11.19 \pm 1.30$ $\mu m/min.$), as it is expected that mitochondria have a higher degree of freedom as far as dynamics is concerned. This is due to the higher interaction of organelles when the parent cells are clustering, i.e., cells are highly interactive when they are in a cluster in comparison to when they are isolated. Figure 6e shows the comparison of the average speed ($\bar{v}$) of mitochondria near the nucleus and the periphery of the cells which are estimated to be $\sim 7.32 \pm 0.74$ $\mu m/min.$ and $\sim 8.62 \pm 1.42$ $\mu m/min.$ respectively. The mean speed of mitochondria is higher at the periphery of the cell than that at the distance close to the nucleus. Again, we estimated the average speed of the mitochondria—comprising of all cases, namely, close to the nucleus, the periphery of the cells (far away from the nucleus), clustered cells, moderately clustered, and isolated— that is found to be 8.08 $\mu m/min.$ with standard deviation of $16.65\%$. This clearly shows that not only cells are not correlated but also mitochondria are not correlated corresponding to the different regions of the cells as well as the nature of the cells. Again, spatio-temporal dynamics of organelles (mitochondria) are similar for similar types of cells but highly uncorrelated with respect to that of different types of cells.

**Table 1:** Comparison of average speed ($\bar{v}$) of mitochondria in the periphery of the cell and near the nucleus.

t-test: Two-Sample Assuming Unequal Variances		
Statistical parameter	In the periph ery of the cell	Near nucleus
Mean	8.62	7.32
Variance	2.09	0.58
Observations	24	17
Hypothesized Mean Difference	0.00000	
df	36	
t Stat	3.72754	
P(T<=t) one-tail	0.00033	
t Critical one-tail	1.68830	
P(T<=t) two-tail	0.00066	
t Critical two-tail	2.02809	

Statistical analysis was performed by the unequal variance t-test (Welch’s test)^31^—which is a statistical test that is used to compare the means of two groups and to determine whether two groups are different from one another—for quantitative characterization of spatio-temporal dynamics (average speed) of organelles (mitochondria) and thus, to check the significance of the difference in average speed of mitochondria near the nucleus and at the peripheral of the cell. The result is presented in Table 1. The obtained *P*-value ($\sim 0.00066$) confirms the statistical significance ($P < 0.05$ which was used to denote significance). Close to the cell nucleus, mitochondria density is observed to be higher than that away from the nucleus and thus, they will be more free to move (at the periphery of the cells). This shows that there exists a higher interaction for organelle (mitochondria) close to the nucleus as it is compared to that in the periphery of the cells. In other words, one may say that the lower interaction of cellular organelles (among themselves and with the nucleus) can be the reason for the higher speed of mitochondria at the periphery of the cells.

**Figure 7:**
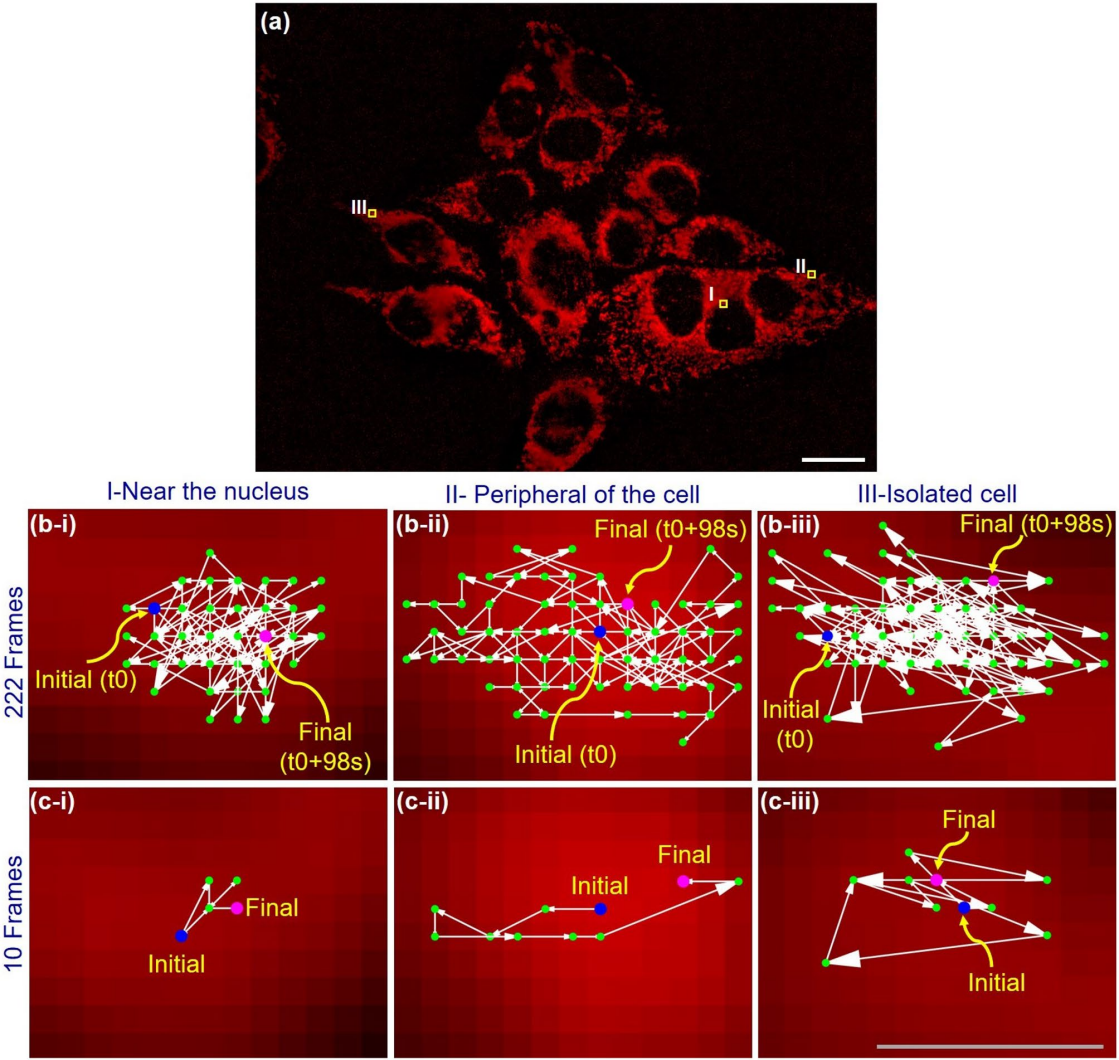
Quantitative evaluation spatio-temporal dynamics (velocity both magnitude and direction) of mitochondria, more specifically, vector representation of time-resolved displacement of three randomly selected mitochondria indicated by marking shown in (**a**). (b-i) to (b-iii) show the displacements for the entire 222 frames while (c-i) to (c-iii) present the displacements corresponding to randomly chosen 10 consecutive frames. In figures ((**b**) and (**c**)), the background images depict the selected mitochondria corresponding to the first frame (at *t*0), i.e., the initial points are marked at the centers of the mitochondria. Scale bar for (**a**): 20 $\mu m$. Scale bar for (**b**) and (**c**): 0.4 $\mu m$.

Figure 7 depicts a typical pictorial representation of the spatio-temporal dynamics of the mitochondria, more specifically, instantaneous displacements or velocities are denoted by vectors. Here, each vector represents the displacement of the mitochondria in sequential (image) frames. In the figure, the length of the arrows and the size of the arrowheads indicate the measures of magnitude of displacement, i.e., the distances through which mitochondria are traversing. The figure depicts the movements of three randomly selected mitochondria (as they are marked in Fig. 7a)—that include one near the nucleus, one from the peripheral of a cell (in a cluster of cells), and one from the peripheral of an isolated cell—for the entire 222 frames (collected at the time interval of 98*s*) are given in Fig.7(b-i) to Fig.7(b-iii). Similarly, Fig.7(c-i) to Fig.7(c-iii) show the mitochondrial movements of the three mitochondria for ten consecutive frames that are randomly picked up (for the sake of better clarity). The starting points of the initial vectors corresponding to *t*0 are marked at the center of the corresponding mitochondria which are in turn represented by the background images in the figures. From the experimental results, the displacements of the mitochondria close to the nucleus (Fig. 7(b-i)) are relatively low in comparison to that of mitochondria at the peripheral of the cell (both for clustered and isolated cells (Fig. 7(b-ii) and (b-iii)). Shortly, we may conclude that mitochondria close to the nucleus move shorter distances or are more highly interacted and thus, tightly bound to the nucleus. We may note that we acquire the sequential images at the same frame rate ($\sim 2.27$ frames/s), i.e., acquisition time period ($\sim 0.44$ s). With the given same acquisition time of image frames, the depicted displacement vectors indicate the corresponding velocity—in both magnitudes (represented by lengths) and directions (represented by arrowheads), and consequently, we may conclude that mitochondria close to the nucleus move at lower speed in comparison to that of mitochondria at peripheral of cells. This may be because of the highly interactive nature of mitochondria close to the nucleus. Again, the mitochondria in isolated cells undergo movement or spatio-temporal dynamics not only at a higher speed but also with higher randomization. This may be because of loose in interaction of the parent cells with other (neighbouring) cells. Lastly, from the figures, it is clear that all of the mitochondria (either isolated or clustered) undergo spatio-temporal dynamics about their thermodynamic equilibrium position.

As illustrated in Fig. 6, lower magnification imaging allows us to observe and study specimens at a wider field of view, encompassing a substantial number of cells. This capability enables the selective identification of specific regions of interest (such as certain clusters of cells/tissue for a selective or focused study). Consequently, correlations among different cells and/or organelles, that include physiological activities (say, spatio-temporal dynamics (velocities), as demonstrated in Fig. 6) and parameters (like the number of clusters and cells per cluster) can be established. This stands in contrast to the conventional techniques where imaging is limited to certain pre-specified cells or organelles, i.e., with a restricted field of view at a given instant. Our proposed dual-arm imaging modality uniquely allows the observation of selective cells or organelles at higher magnification or resolution, akin to conventional techniques, without sacrificing generality. Prior to our studies, the simultaneous acquisition of multiple image frames—at different magnifications, spatial resolutions, and fields of view— presented a technological challenge, even with conventional fluorescence microscopy.

Our study further demonstrates the quantitative characterization of spatio-temporal dynamics (velocity, both amplitude and direction) of organelles (mitochondria) and their mutual correlations. Notably, mitochondria close to the nucleus (or in clustered cells) exhibit a lower degree of freedom compared to those at the cellular periphery (or isolated cells), as highlighted in the Abstract. In addition to velocity/speed, it will be interesting to look at other parameters like mitochondrial membrane potential which influence the energy production capacity of mitochondria various across different clusters of cells or within cell populations^32^. Consequently, the quantitative analysis of mitochondrial membrane potential in different cell clusters holds valuable information, especially in tissue samples where variations in mitochondrial movement and energy production capacity exist across cell populations. Tissues exhibit larger heterogeneity in their cell population both in terms of density of cells as well as type of cells. Hence, it is imperative to study how the nearby cells influence the activity of internal organelles like mitochondria. Lower magnification aids in classification like relative positioning and density, while higher magnification provides the necessary resolution for quantitative measurements. Furthermore, our microscope facilitates studying interactions between mitochondria and the microbiome. Mitochondria, with evolutionary origins linked to bacteria, exhibit similarities with certain bacterial structures. Investigating these interactions in a larger context may provide insights into the co-evolution and symbiotic relationships between mitochondria and bacteria. The movement and energy parameters of mitochondria can be measured while cells interact with surrounding microbes. It will be interesting to have quantifiable data on how the position of microbes influences various organelles in the cells^33–35^. Another intriguing phenomenon is organelle transfer between cells like mitochondrial transfer, and it would be fascinating to quantify various parameters during this process. Questions about which mitochondria are chosen for transfer from donor to recipient cells, their movement relative to the rest of the mitochondrial population, and their energy levels could be answered through a holistic view provided by lower FOV and a magnified view for accurate measurement. While these experiments were beyond the scope of this manuscript due to various constraints, the potential for future investigations is evident^36,37^.

### Multi-spectral imaging

#### Mouse embryo

**Figure 8:**
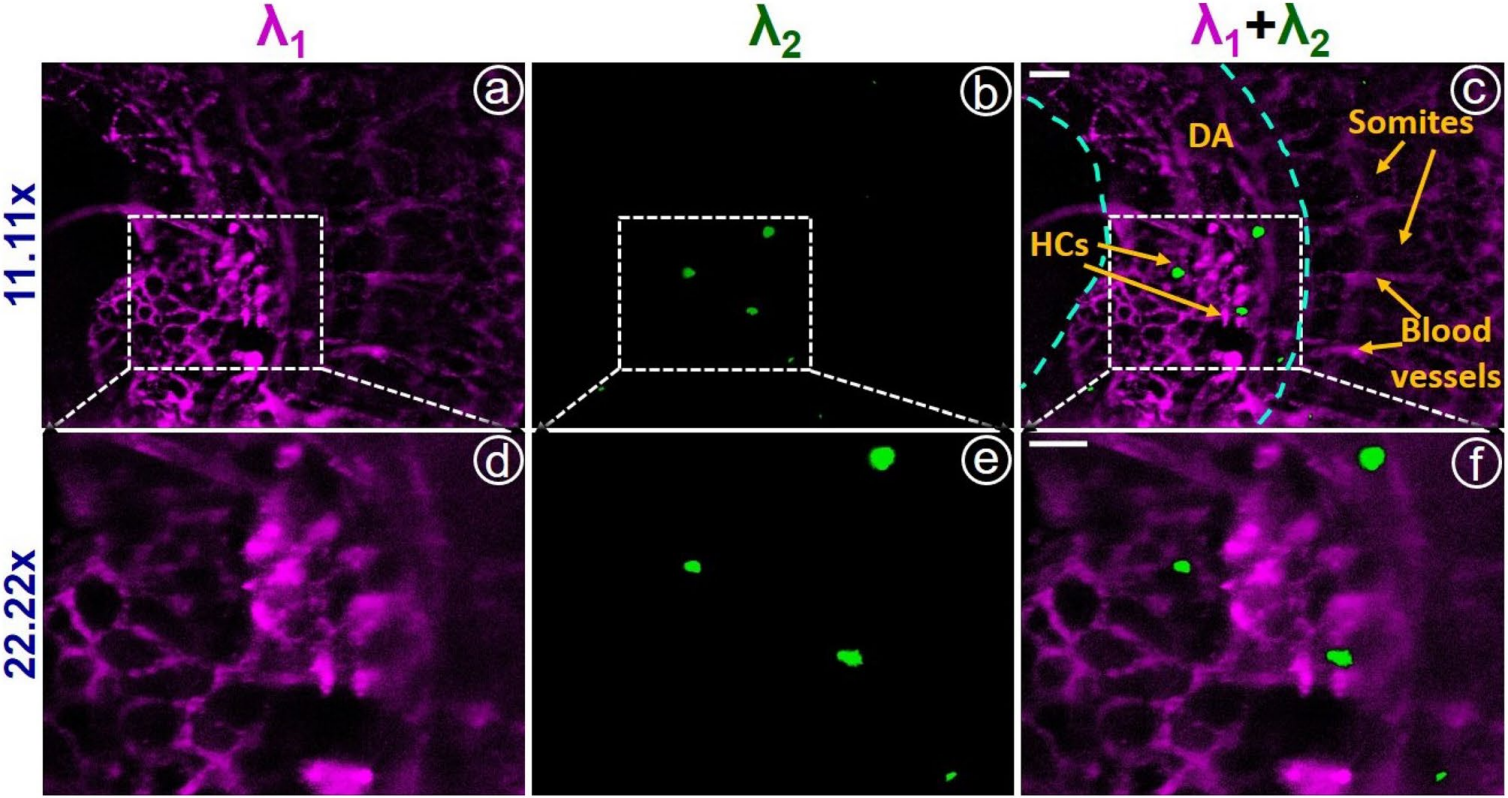
(**a**) and (**b**) shows images (obtained at $11.11\times$) of AF594 tagged CD31 positive endothelial cells and AF488 tagged HCs respectively. (**c**) shows the merged or fused image for (**a**) and (**b**). (**d**), (**e**), and (**f**) show the corresponding images obtained with $22.22\times$ magnification. DA marks the dorsal aorta. Arrowheads mark HCs, somites, and blood vessels/vascular networks. Scale bar: 50 $\mu m$.

In the E10.5 mouse embryo, Hematopoietic cells (HCs) are seen associated with the endothelial lining of major blood vessels such as the dorsal aorta (DA). Here, we mainly focus on such regions to locate the HCs. The laser was tuned at an optical wavelength ($\lambda _{1}=594$ nm) to image endothelial cells. Images are recorded simultaneously at $11.11\times$ and $22.22\times$ magnifications and they are depicted in Fig. 8a and d respectively. Further, the images of HCs are acquired by tuning the wavelength at $\lambda _{2}=488$ nm. Figure 8b and e show the images obtained at $11.11\times$ and $22.22\times$ magnifications respectively. The individual images of endothelial cells and HCs are merged to get the multi-spectral image at both magnifications, which are given in Fig. 8c and f. From the merged image, we can locate HCs within the DA, in which the DA, somites, and vascular networks are indicated by arrowheads in Fig. 8a.

#### HeLa cells

**Figure 9:**
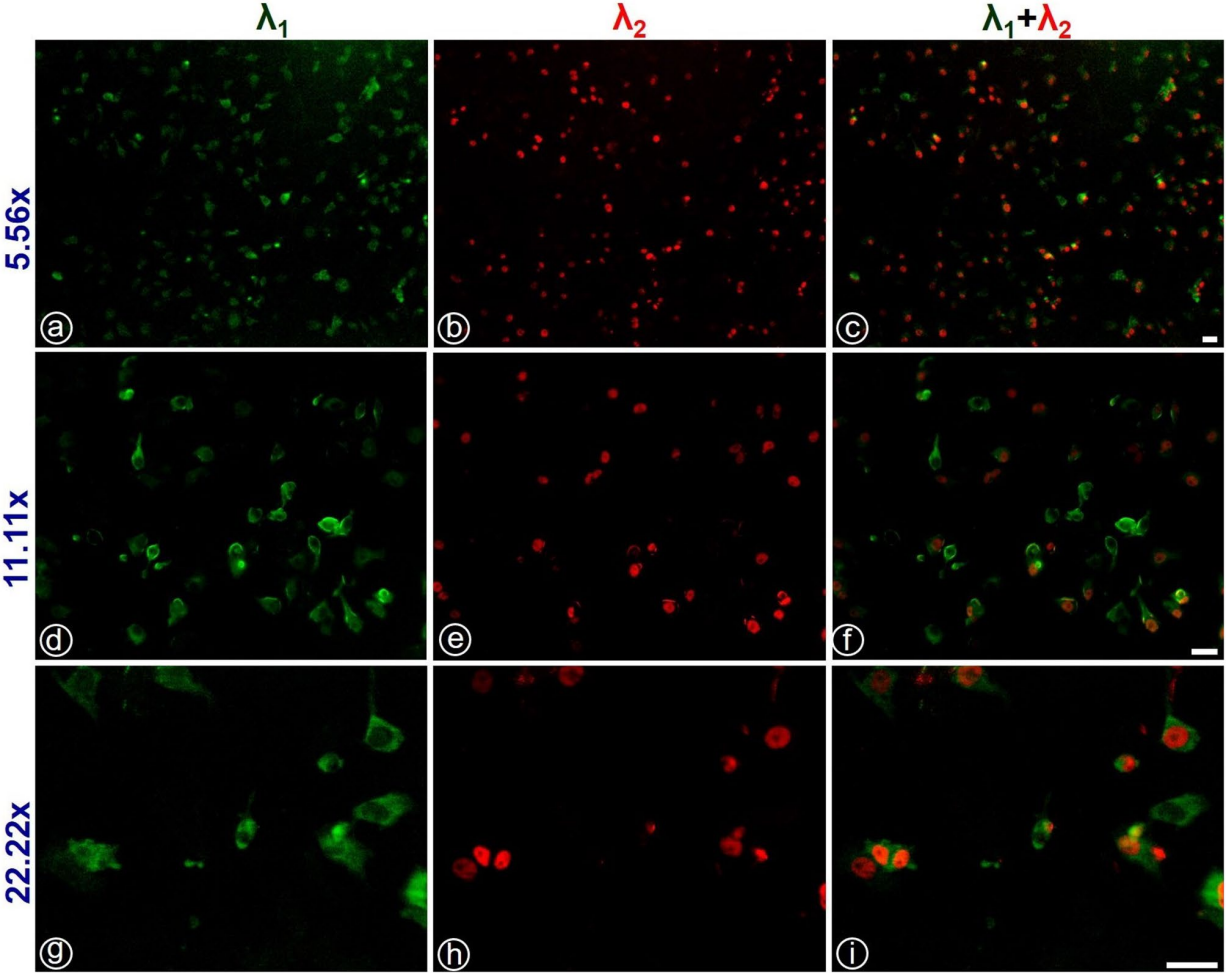
(**a**), (**d**), and (**g**) show images of microtubules, and (**b**), (**e**), and (**h**) show the nucleus of HeLa cells for the magnification $5.56\times$, $11.11\times$, and $22.22\times$ respectively. (**c**), (**f**), and (**i**) are the merged images of the nucleus and microtubule. Scale bar: 40 $\mu m$.

Figure 9 shows the multispectral imaging of HeLa cells at three different magnifications, namely, $5.56\times$, $11.11\times$, and $22.22\times$. The laser source was first tuned to a wavelength $\lambda _{1}=488$ nm to image the microtubules and then tuned to $\lambda _{2}=590$ nm to image the nuclei of the cell line. The individually obtained images were merged (as mentioned above) to obtain the complete image of HeLa cells. The localization of nuclei and microtubules is well observable from the multispectral image. This is to note that one can select optical excitation at any wavelength in the range from $420-670$ nm (even though the experiments were conducted only for two wavelengths in this present study). Again, our proposed M$\lambda$-sMx-SPIM imaging technology facilitates giving any optical excitation wavelength by the change in the tuneable pulsed laser source.

## Discussion

Conclusively, we may draw the technical novelties of the studies presented in this article as: (i) feasibility for studying spatio-temporal dynamics of physiological activities of alive biological specimens (over entire volume not only for a particular section), i.e., 4D, (ii) to acquire two image frames of a slice or section of a larger biological specimen ($\sim$ mm) with different FOVs at different resolutions or magnifications simultaneously in real-time, (iii) to resolve or address the longstanding challenges of housing multiple light sources with operating wavelength over a wide range $\sim 420-670$ nm which is in contrast to a limited number of optical wavelengths ($\sim 6$ at the maximum till date) as it is in the case of the conventional CW laser source-based microscopy (in general) and light sheet fluorescence microscopy (LSFM) (in particular), (iv) quantitative characterization of spatio-temporal dynamics of physiological activities of organelles and their mutual correlationships (velocity (amplitude as well as direction) (for mitochondria), and development and growth of all tracheal branches of embryonic development (Drosophila) over an entire period $\sim 95$ min). The experimental results demonstrate that the study is of great scientific impact both from the aspects of technology and biological sciences.

One of the disadvantages of employing OPO tuneable laser source (instead of CW laser sources) is that OPO laser is relatively expensive. However, its potential technical applications are not comparable (or unparalleled) and not achievable with that of CW laser sources. More elaborately, physiological activities of biological specimens recoverable with the conventional techniques are limited and they are restricted by the limited number of photo-excitation wavelengths (say, 6 at the maximum till date). This pertaining technical challenge is successfully addressed with our proposed tuneable pulsed laser-based LSFM that gives any photo-excitation wavelength over an entire range of 420-670nm. One may draw an additional drawback that OPO tuneable laser source gives out one optical wavelength at a time (but sequentially with the temporal resolution being determined by pulse-repetition-frequency (PRF) which is $\sim 100$ Hz in our present study, i.e., temporal resolution $\sim 10$ ms). Typically, in raster-scanning-based confocal microscopy, which remains as the gold-standard imaging technique for biological applications, image acquisition-time is of the order of minutes. The argument implies that tuning of optical wavelength or photo-excitation at temporal resolution $\sim 10$ ms is several orders less than the conventional image acquisition time-scale ($\sim$ min) and thus, can be relatively considered as simultaneous photo-excitation. On the other hand, in the case of the conventional CW-based LSFM, the image frame-rate is determined by the frame rate of the optical camera (as an optical sensor array) which is typically limited to $\sim 100-200$ frames/s against our proposed PRF of tuneable pulsed laser source at 100 Hz. This demonstrates that our proposed tuneable photo-excitation can be relatively considered as simultaneous photo-excitation for multiple fluorophores (even though we may assume it to be simultaneous).

In summary, we report the development of a unique microscopy imaging technology (M$\lambda$-sMx-SPIM) that addresses certain technological challenges: (i) obtaining separate image frames of a specimen at different resolutions and field of views simultaneously in real-time, (ii) imaging of entire volume of larger and alive biological specimen (size $\sim$ mm), (iii) any optical excitation wavelength in the range from $420-670$ nm, and (iv) to study spatio-temporal dynamics of physiological activities of biological (alive) specimens in a wide range of organism to cellular organelles. Experimental validation studies were carried out in various and diversified biological specimens that include *Drosophila melanogaster*, mouse embryo, and HeLa cells. This imaging technique can also be used to study various other (alive or fixed) biological systems at organism, cellular, and sub-cellular levels and thus, it will be of significant impacts to address various biological questions. The study will be of great impact not only to the imaging community but also to biological understanding and its application (including regenerative medicine).

## Methods

### Experimental Set-up and Procedure

**Figure 10:**
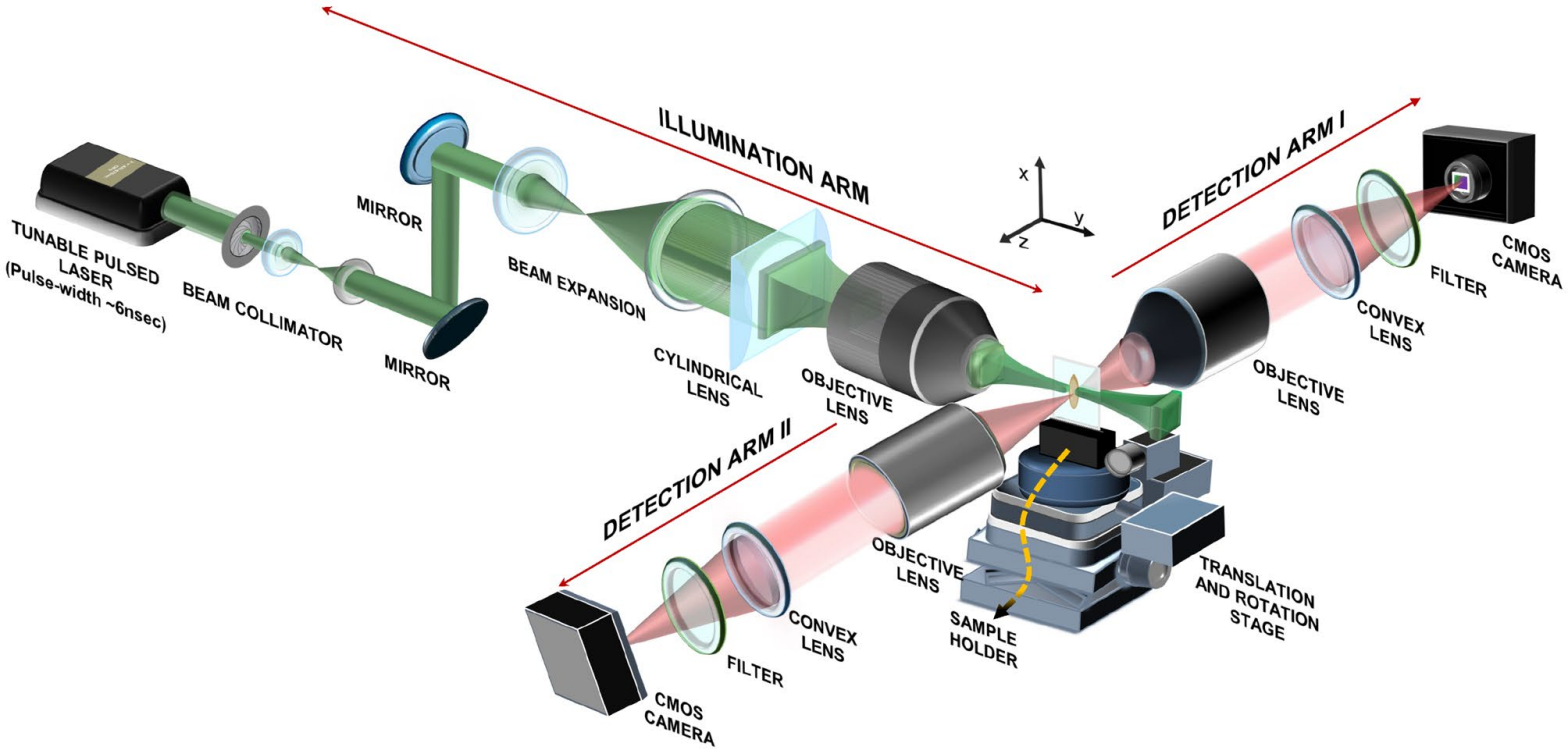
Schematic diagram of the experimental system (using our M$\lambda$-sMx-SPIM).

**Figure 11:**
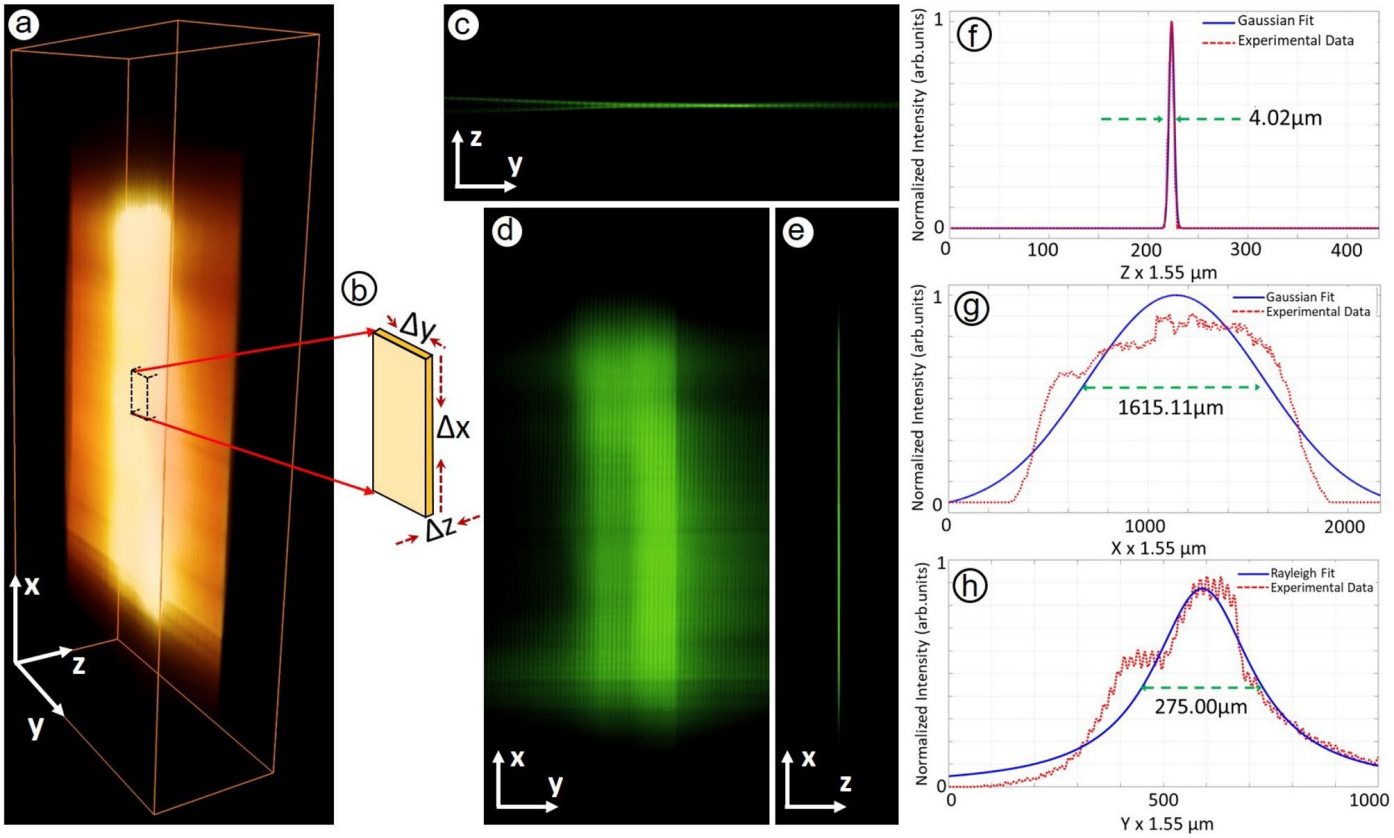
Light sheet characterization of the M$\lambda$-sMx-SPIM system. (**a**) 3D view of the light sheet. (**b**) A cropped image of 3D representation near the focal zone of the light sheet. 2D cross-sectional views of the light sheet in the three separate and mutually perpendicular planes (**c**) yz-plane at x = 945, (**d**) xy-plane at z = 223, and (**e**) xz-plane at y = 610. Line plots of normalized light intensity distribution along the z-axis (**f**), and x-axis (**g**), both fitted with Gaussian function and along the y-axis (**h**), fitted with Rayleigh’s function.

Figure 10 depicts a schematic diagram of the experimental system. A thin sheet of light (thickness $\sim \mu m$) was used to selectively induce photo-excitation of target specific fluorescence dyes being distributed over a thin section of the specimen of interest for imaging and the subsequent photo-emission light resulted due to the fluorescence effect is acquired in parallel by optical sensor array (say, the optical camera kept in orthogonal configuration with respect to light illumination (see Fig. 10)) to generate a 2D image in real-time. In our home-built M$\lambda$-sMX-SPIM (Fig.10) imaging system, we fitted two detection arms (instead of a single arm as it is in the case of the conventional LSFM) that are co-aligned along a horizontal axis (but orthogonal to the axis of optical illumination beam). The two detection arms are fitted separately with optical objective lenses of different magnification powers and thus, can be controlled independently while focusing both of the objective lenses to a single imaging plane. This unique optical configuration enables to acquire two image frames of a single imaging plane of the specimen of interest at different magnification levels, image resolution, and FOVs. One can vary the obtainable magnifications by changing the objective lenses in the detection arms (at ease). In addition, this facilitates the simultaneous acquisition of two separate 2D images from two separate sections or sub-sections of the imaging plane in real-time. We can control the movement of the mechanical stages (for the optical illumination arm as well as the two detection arms) independently in all of the three translational axes (*x*, *y*, and *z*). On the other hand, the sample holder was fitted to a mechanical system constituted by three individual motion-controlling stepper motors that enable us to control the movement (both rotation and translation) of the imaging specimen at sub-microscopic step-size or step-resolution. In this way, we can obtain sequential images—corresponding to the microscopic or sub-microscopic relative motion—and thus, reconstruct 3D images (through post-processing). We can achieve a relatively high frame rate that is dictated by the frame rate of the camera adapted as an optical sensor array in the detection arms (Arm I and Arm II). In this present study, we employed CMOS camera (BFS-U3-13Y3M-C) with: (i) frame rate $\sim 170$ frames/s. that corresponds to image acquisition time $\sim 6 ms$., (ii) pixel resolution or density $\sim 1280 x 1024$ that serves as an array of sensors arranged in a rectangular grid. The individual sensor elements in the array acquire the boundary data simultaneously at a time. In this way, the image frame rate is not related to the physical dimension of imaging of interest and/or pixel density which is in contrast to the case of raster-scanning-based microscopy (including confocal microscopy) where the obtainable image frame rate is directly proportional to the area of scanning. In addition, we employed a tuneable pulsed laser OPO laser source (SpitLight OPO EVO S, Innolas Lasers, Germany; pulse width $\sim 6$ ns; pulse repetition frequency (PRF) that can be varied from 1 to 100 frames/s; operating wavelength ($\lambda$) $\sim 420-670$ nm with the wavelength tuning step size $\sim nm$) in the illumination arm. This tuneable pulsed laser serves a number of CW laser sources (not just 3-4 as it is done in the conventional LSFM). Shortly, our home-built sMx-SPIM system is capable of real-time imaging samples at various magnifications and fields of view. It also facilitates adequate acquisition speed to record biological processes or activities ($\sim$ ms) over a longer period of time or duration (hours or days). Figure 11a depicts a 3D view of the light sheet (as it was done in^18^) while Fig. 11b shows the 3D representation near the focal zone of the light sheet over which the light sheet has a uniform thickness. Figures 11c, 11d, and 11e present 2D cross-sectional views of the light sheet in the three separate and mutually perpendicular planes (say, yz-, xy-, and xz-planes) corresponding to x = 945, z = 223, and y = 610 respectively. Again, to characterize the obtainable axial resolution (along the z-axis) and the extent of the field of view (along the x-axis and y-axis), we present a line plot depicting the distribution of the detected light intensity along the z-axis (Fig. 11f), x-axis (Fig. 11g), and y-axis (Fig. 11h) at z=223, y=610, and x= 945 respectively. As it was done in our previous study^18^, we undertake curve-fitting by using the Gaussian function for the line-plot along the z-axis (to estimate the thickness, i.e., the axial resolution of the light sheet) and along the x-axis (to find FOV), and Rayleigh’s function for the line-plot along the y-axis (to estimate depth-of-field). They are estimated to be $\sim 4.02$ $\mu m$, $\sim 1615.11$ $\mu m$ and $\sim 275.00$ $\mu m$ respectively. The spatial resolution obtainable was estimated to be $\sim 0.31$ $\mu m$ (for $11.11\times$), $\sim 0.16$ $\mu m$ (for $22.22\times$), and $\sim 0.08$ $\mu m$ (for $44.44\times$) from the aspect of the experimental resolution limit^18,19^. However, the resolution is limited by the wave theory or the Rayleigh’s criterion of diffraction limit, and thus the achievable lateral resolution is estimated to be 0.865 $\mu m$. More details of axial and lateral resolutions of the microscopy system under different magnifications are discussed in Supplementary 3. In this study, we demonstrate observation and recording of the development of the tracheal system (of *Drosophila melanogaster*) in real-time at two different magnifications over an extended period of $\sim 95$ min This is to note that, from mechanical aspects, the imaging unit is mandated to be isolated properly from the ambient mechanical movement. For this, we kept the imaging unit on the top of a vibration-free optical table.

During the experiments, one needs to take utmost care for proper handling of the alive imaging specimens (both for *Drosophila* and mitochondria unlike the case of fixed samples (say, mouse embryo)). One needs close and continuous monitoring of the specimen being alive or dead over the entire tenure of the experiments. This is to note that our system demands vertical mounting of the imaging specimen for which we developed a vertical mounting mechanism. For post-processing of the collected data or sequential images, we adopted various softwares (MATLAB, image-J, and Amira) and algorithms (median filtering, thresholding, band-pass (frequency), tracking, etc.).

### Sample Preparation

#### Drosophila *embryo*

The breathless (*btl*) gene is essential for tracheal branching^38^. To visualize the tracheal structures within the *Drosophila melanogaster* embryo from the earliest stages of its development, we set up a cross between *btl*-Gal4,UAS-*gfp*/CyOGFP virgins and UAS-*gfp* males (F:M=2:1), and from the F1 progeny, only the embryos with the following combination were used for imaging *btl*-G4,UAS-*gfp*>UAS-*gfp*. For details, one may refer to the reported studies^5,39–41^. For proper staging of the embryos, the flies were allowed to lay eggs on apple-juice agar plates for 2 h. The embryos were incubated at $25^\circ$C for 5 h (for late Stage 10 embryos) or 9 h (for late Stage 12/early Stage 13 embryos) and 13 h (for Stage 15 embryos). Embryos with endogenous tracheal GFP expression were transferred onto a microscope slide with double-sided tape. We gently rolled the embryo until the embryo popped out of the chorion. Then, the dechorionated embryos were placed on a soft agar-coated coverslip in a desired orientation. We applied a thin coating of halocarbon oil on the embryos and placed the cover slip on the sample holder (mentioned above) for the experiments.

Sample Standardization:

Different sample preparation approaches were standardized for compatibility with our home-built (M$\lambda$-sMx-SPIM) microscopy system. Agar-coated capillary tube based method^42^, where the embryos are fitted within a capillary tube containing agar, remains one of the most commonly used methods. The method suffers a serious drawback. In this method, the working distance between the sample and the lens was found to be very limited, thereby, resulting in image blurring. We found agar-coated cover-slip—among various other approaches including agar-coated glass slide, double-sided tape-coated cover slip, and agar-coated cover slip—to be the best suited or optimal sample preparation method (for our present study). The thin agar coating minimized the reflection during imaging or experiments, but it could not sustain the sample in live conditions for more than 45 min. To overcome this, we applied a thin layer of halo-carbon oil to the sample that maintains the sample alive for a comparatively longer duration (2–3 h).

#### HeLa cells

For dynamical studies of mitochondria:

HeLa cells obtained from American Type Culture Collection (ATCC) were cultured and stained with mitotracker as described in our previously reported study^43^. To mount the sample vertically to be adaptable in our home-built (M$\lambda$-sMx-SPIM) microscopy system, the cell was cultured in a glass bottom dish (Cell Vis, D35-20-1.5-N). After 18–24 h of plating and staining with MitoTracker^TM^ Red CMXRos, the bottom was filled with a small amount of DMEM solution and then, sealed with a cover slip using clear nail polish. The samples were immediately used for imaging. This is to note that the glass bottom was kept vertically by using a custom-made mounting base on the sample mounting stage (mentioned above).

For the multispectral imaging:

The nuclei of HeLa cells (obtained from American Type Culture Collection (ATCC)) were stained with H2A-mcherry and microtubules were stained with Tubulin-GFP. The cells were grown on a cover slip in a culture dish for two days. DMEM media supplemented with 10% fetal bovine serum. Penicillin and streptomycin were used as the growth media. The cells were incubated in a humidified 5% CO2 incubator at $37^\circ$C temperature. After 24 h, the cover slip having cells was mounted on a custom-designed mounting stage in a vertical orientation as mentioned above.

#### Mouse embryo

In this project, whole mouse embryos at embryonic day (E10.5) were employed as samples. The embryos were harvested, dissected and fixed in 2% PFA. Following fixation, the embryos were dehydrated using methanol gradient and stored at $-20^\circ$C. Prior to immunostaining, the embryos were trimmed and rehydrated using a series of methanol and PBS. Following serum/BSA blocking, the embryos were stained with antibodies against c-kit and CD31. Secondary antibodies with AF488 and AF594 respectively were used. The embryos after washing and dehydration, were cleared and mounted on a coverslip with fastwell with a 1:2 mix of benzyl alcohol and benzyl benzoate (BABB). Stained embryos were stored at room temperature till imaging.

### Statistical Analysis

The unequal variance t-test (Welch’s test) was used for statistical analysis. The *P* value (*P*) $< 0.05$ was used to denote the significance. All statistical analyses were performed in Windows-Excel (2019).

### Supplementary Information

Supplementary Information 1.

Supplementary Information 2.

Supplementary Information 3.

Supplementary Information 4.

Supplementary Information 5.

## Data Availability

The datasets used and/or analysed during the current study available from the corresponding author on reasonable request.
