# Supplementary material for: Real-time study of spatio-temporal dynamics (4D) of physiological activities in alive biological specimens with different FOVs and resolutions simultaneously
Source: Sci Rep. 2024 Feb 12;14:3542. doi: 10.1038/s41598-024-52152-x (PMC10861542; doi:10.1038/s41598-024-52152-x)
Supplement: Supplementary file 1 — Supplementary Information 1. [file 41598_2024_52152_MOESM1_ESM.pdf]

Supplementary

Supplementary 1: Development of Tracheal System in *Drosophila Melanogaster*

In insects, such as fruit flies (*Drosophila melanogaster*), the circulatory system is of open type. In order to ensure quick and efficient delivery of oxygen to all parts or tissues within the body, the fruit fly has in place a unique network of progressively branching and interconnected tubes (known as the tracheal system). Shortly, the pathophysiological activities of tissues in each and every section and sub-section of the organism (including the innermost regions of tissue) are controlled and regulated by the tracheal system. In the fruit fly larvae, the trachea is constituted by two large (multi-cellular) tubes − called dorsal trunks − that run throughout the entire length of the body connecting to the exterior or external environment by means of anterior and posterior pairs of spiracles. Each DT gives rise to ten individual primary branches with each primary branch controlling and/or regulating the supply of one-half of each body segment. Each primary branch further gives rise to several secondary and tertiary (terminal) branches that innervate specific tissues in their respective segments. All tracheal branches ultimately terminate in single cells − known as tracheal terminal cells (TTCs) − that produce finger-like extensions of its cell body innervating the interior-most regions of tissue. The trachea arises from 10 cell clusters of ectodermal origin − the outermost layer of the three primary germ layers formed in early embryonic development − on each side of the embryo that appear around Stage 5 of embryonic development (∼ 3hrs after egg laying)[1]. Each cluster of cells gives rise to a portion of the dorsal trunk (also known as dorsal longitudinal trunk (DLT)) and the transverse connective (TC). The TC further buds off to produce a spiracular branch (SB), a visceral branch (VB), an anterior lateral connective branch (aLLT), a posterior lateral connective branch (pLLT), and ganglionic branches (GB). At Stage 14 (∼ 10hrs after egg laying) of embryonic development, the individual dorsal trunks of each segment fuse with each other to form a continuous dorsal trunk that also connects to the dorsal trunk on the other side of the body through dorsal connectives. At the same time, the aLLT of each hemi-segment anastomoses with the pLLT of the adjacent hemi-segment to complete the lateral tracheal trunk. The terminal branching starts appearing by Stage 16 of embryonic development and continues to be it continues to change or re-model throughout the life of the larvae[1,2]. Such understanding of developmental biology was concluded from the study using state-of-the-art microscopic imaging technology (more particularly, wide-field and confocal microscopy). In addition, for the reported studies, the specimens are fixed not in alive condition. Shortly, the present understanding of developmental biology is drawn from observation and studies in fixed biological samples (not alive). Here, in this article, we report the technological potentiality of an emerging imaging modality (namely, light sheet fluorescence microscopy (LSFM)) for the real-time observations and studies of the entire sequential biological procedures for the development of tracheal system with specimen (embryos) in alive and fully intact condition. Again, our home-built LSFM imaging system enables imaging of two individual frames of a single section/slice of a given specimen at different magnification levels and at different fields of view simultaneously. This feature permits the investigation of the correlations between the physiological and functional activities of microscopic structures, whether cellular or subcellular, and their immediate surroundings or other specimen elements. Unfortunately, state-of-the-art imaging technologies (in general) and SPIM (in particular) still fail to address the challenge of obtaining images of a specimen at different magnification levels simultaneously at a given instant of time but are mandated primarily for visualization and study of spatio-temporal dynamics of biological specimen of interest at various levels of spatial resolution and magnification (including molecular[3,4] and organelle (at Å scale), cellular (at sub-microscopic level)[5–7], tissue (at microscopic level), organs and organism (at mm to cms)[8,9]). In other words, a variety of imaging techniques with various spatial resolutions and magnifications are necessary for understanding the complex biology of living things as well as the onset and progression of illnesses.[10]. The experimental results of this validation study demonstrate the promising technical features of our Mλ-sMx-SPIM imaging technology for the study of developmental biology and its applications.

Supplementary 2: Inherent Trade-off Between the Achievable Spatial Resolution (or Magnification) and Field of View (FOV) in an Imaging System

In the reported developmental biology studies, the adopted LSFM imaging technologies enable to give a single image frame at a given instant of time. For a given sensor array of limited physical extent, there exists an inherent trade-off between the obtainable magnification (or spatial resolution) and FOV. This implies that, for an optical system, the physical dimension of the image (in the image plane) corresponding to an object plane increases with an increase in the magnification power of the (optical) imaging system. Thus, for a given physical dimension of the sensor array situated at the image plane, the physical extent of the object scene (known as FOV) that can be perceived through the sensor array is reduced with an increase in magnification or resolving power[11,12]. In other words, an optical microscopy imaging system with higher magnification power − which is suitable for observation, analysis, and study of microscopic and sub-microscopic (say, cellular, molecular, and organelles) details of biological specimen − is not of merit for observing entire biological specimen (of dimension *mm*) and vice-versa[5]. Due to this technical limitations, we are unable to simultaneously (or hand-in-hand) observe sub-microscopic features and the overall structure of the biological specimen. From the aspects of developmental biology, the reported studies fail to observe the structures and their changes at sub-microscopic levels occurring simultaneously in the various sections and/or

sub-sections during the developmental process and thus, their correlationships. In order to study the interconnected dynamics at various scales of development (including everything from intracellular interactions to effects on the whole embryo), a view of the entire biological specimen is required during embryonic development, which is characterized by shape transformation, migration, and remodeling of tissues or organelles.[8].

Supplementary 3: Axial and Lateral Resolution Under Different Magnification

In LSFM imaging modality, obtainable axial resolution is solely determined by the illumination arm, more particularly, magnifying power of the objective lenses adapted in the illumination arm (see Fig. 11 in main article). Elaborately, in LSFM as it is explained above, a given specimen is virtually (not physically) sliced down by selectively illuminating only a specific section of the specimen (not the whole specimen). This is achieved by employing tight focusing optical system comprising of an optical cylindrical lens and a spherical conversing lens. The resolution, at which the imaging specimen can be sliced down, is characterized by the thickness of the light sheet. This is what we call as axial resolution in LSFM imaging modality. The focal spot size (or thickness of light in the case of cylindrical lens) is characterized by the magnifying power of the corresponding lens, i.e., with an increase in magnifying power (or numerical aperture) of the lens, the obtainable focal spot size as well as focal-zone length are reduced. Typically, by definition, focal-zone length means the physical extent over which the spatial distribution of optical intensity can considered to be uniform (see Fig. 11 in main article). In true sense, in LSFM, this is the physical extent that is considered for imaging in a given specimen, i.e., the physically accessible FOV is characterized by the obtainable focal-zone length of the lens combination in the illumination arm. In addition, the physical dimension (not only pixel or sensor density) of optical sensor array of the cameras – attached in the detection arms (see Fig. 10 in main article) – is limited. In this way, for a particular magnification power of the objective lenses attached in the detection arms, the obtainable FOV is restricted. Shortly, in LSFM, obtainable FOV is determined by either the focal-zone length or FOV achievable with the detection arm whichever is smaller. In our present study, obtainable FOV is determined by the FOV associated with the detection arm. On the other hands, by the lateral resolution, we mean to say resolution obtainable along the direction perpendicular to the axial direction. This is determined by the pixel dimension of the camera attached to the detection arms. This is worth to note that the above argument is not contradictory to the wave theory of light – which determines the maximum obtainable (spatial) resolutions as, $\alpha = \frac{\lambda}{2NA} \approx \frac{\lambda}{2}$ (where λ is the wavelength and NA is the numerical aperture)[12,13]. For our study, α is estimated to be 240 nm (for $\lambda = 480$ nm) which is of the order of magnitude less than that of the above-mentioned lens parameters (say, light sheet thickness and pixel size).

Supplementary 4: Photo-damage

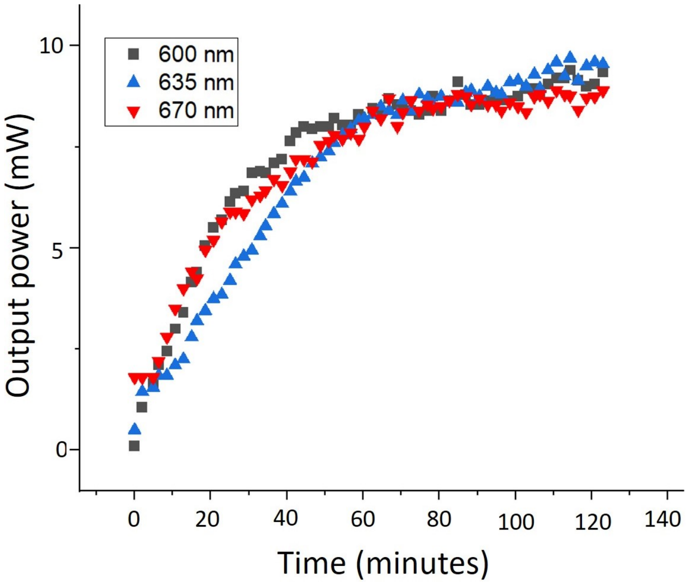

Figure S1-I: Measured optical power of the pulsed laser beam for three different wavelengths with respect to the duration of on-time.

Figure S1-I depicts the measured optical intensity of the pulsed laser beam with respect to the duration of on-time where optical power is employed as experimental parameter. For this experimental study, we employed optical power meter (OPHIR StarBright – 10A-V1.1ROHS). From the figure, it is clear that the optical beam attains stability over an interval of 1hr of

on-time. This is to note that we employed this stable optical intensity in our experiments. During the experiments, the imaging samples (namely, Drosophila melanogaster and HeLa cells) were kept alive. To study the photobleaching throughout the 95 minutes imaging of Drosophila, the fluorescence intensity variation of three different tracheal branches, namely, DLT, TC07, and TC06, were analysed. The graphical plot of the normalized fluorescence intensity per unit pixel vs. time is given in Supplementary Fig. S1-II). The fluorescence intensity was linearly fitted with time and there is an average decrement of $8.41 \pm 0.02\%$ of fluorescent intensity over 95 minutes. The intensity variation is linear over the time of imaging. Conclusively, in our standardization study of photo-damage to the specimen, the photo-damage over the time interval of our experiments (say, $\sim$ 95 minutes) with the optical power ($\sim$ 10 mW) is relatively low. We found that, with an increase of optical intensity, the time duration of sample exposure to the optical beam is required to be reduced so as to avoid the photo-damage of the alive sample as well as photo-bleaching.

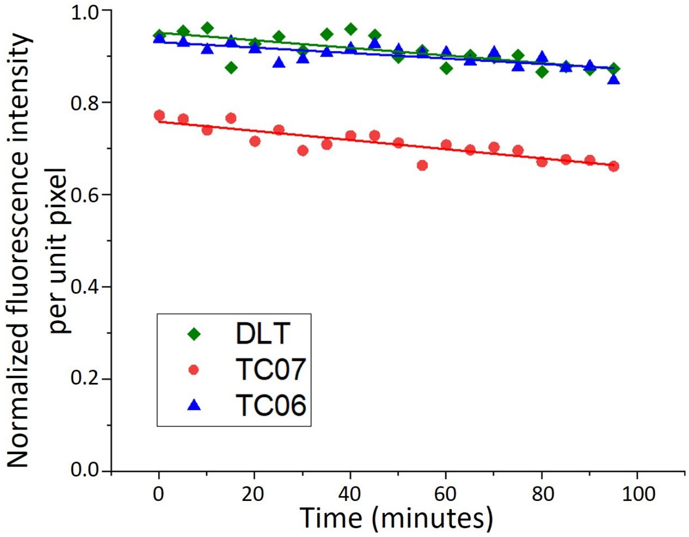

Figure S1-II: Photobleaching over 95 minutes imaging of *Drosophila* tracheal branches.

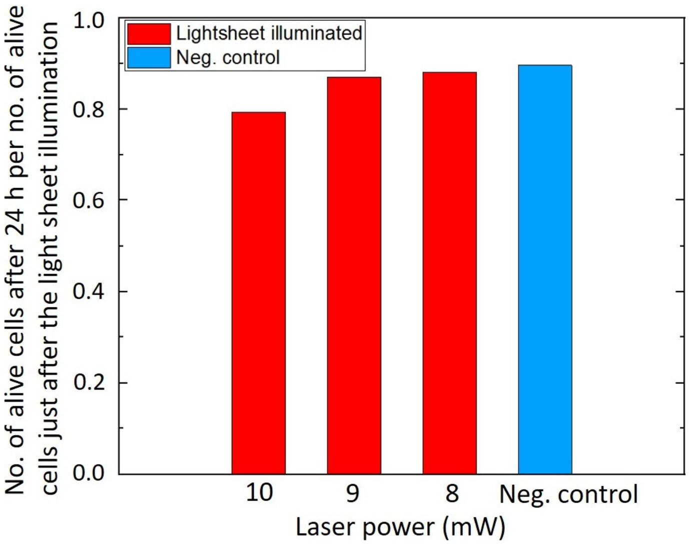

Figure S1-III: Long term viability imaging after light sheet illumination. Maximum laser power is 10 mW.

To find the long-term viability of imaging of the samples after illumination at different laser powers, A549 cells (plated at 40% confluency) were illuminated with light sheet (after 24 hr of plating) over $\sim$ 15 minutes duration (on a single frame) continuously in the Mλ-sMx-SPIM and the number of alive cells were estimated just after the illumination of light sheet and after 24 hr using phase contrast microscopy (Zeiss PrimoVert Inverted Phase Contrast Microscope). The experiment was repeated over three different laser powers (say, 100%, 90% and 80% where the maximum power of the laser is 10 mW, i.e.,

the measured optical power is 10 mW, 9mW, and 8 mW respectively). The experimental results are provided separately in Supplementary Fig. S1-III). The number of alive cells is reduced comparing to the initial number of cells (just after the light sheet illumination) for all the optical powers and the negative control (which is not illuminated with light sheet). This may be because of the fact that the microscopy system is not currently kept inside the incubator. But, from the figure, it is visible that the long-term viability of imaging is reduced for higher laser power. Similarly, in comparison with the negative control, the reduction of alive cells is not so high. Hence, we can conclude that the imaging has long term viability for the laser power less than $\sim$ 10 mW.

Supplementary 5: Image Processing to Improve Image Quality

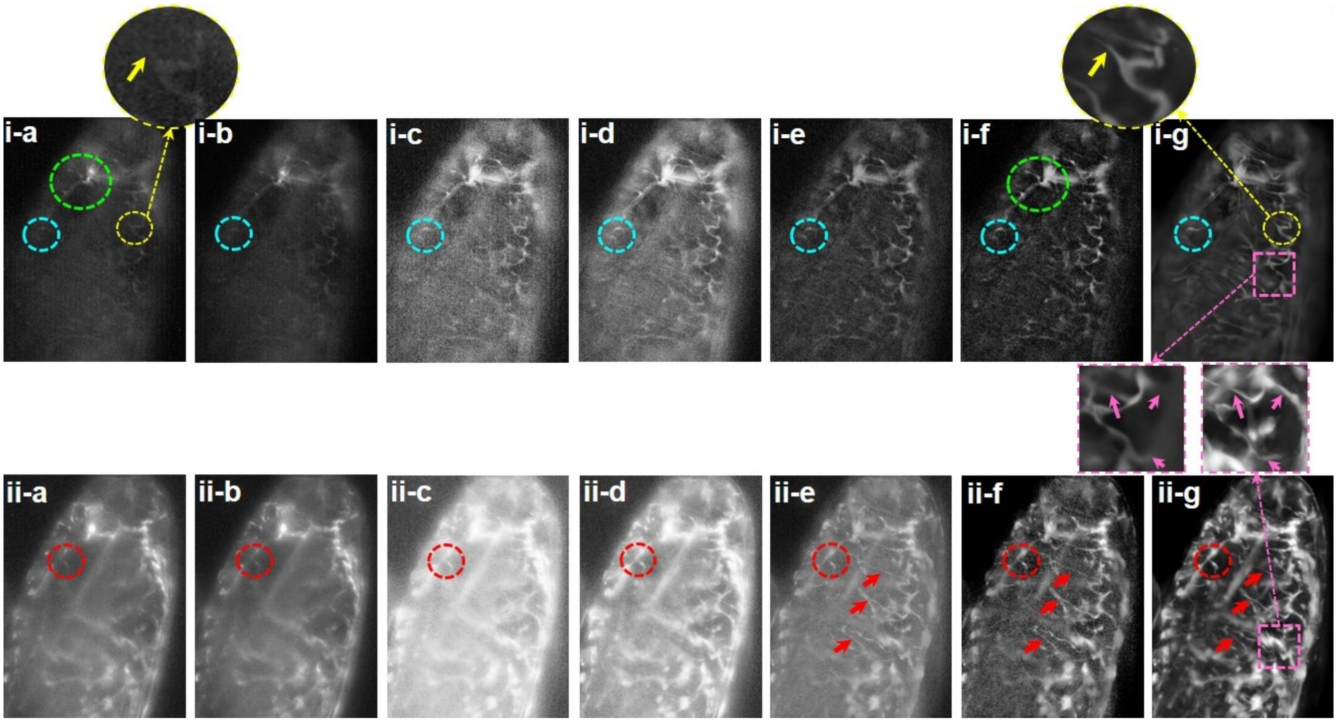

Figure S2: Steps of image processing for a single frame, (i-a) unprocessed image, (i-b) background subtracted (MATLAB), (i-c) CLAHE (Contrast Limited Adaptive Histogram Equalization) image (MATLAB), (i-d) median filtered image (MATLAB), (i-e) background subtracted image (ImageJ), (i-f) brightness and contrast enhanced image (ImageJ), and (i-g) noise reduction using denoise AI algorithm (Visual Paradigm software). MAP images, with projection being undertaken in *xy*-plane, corresponding to each step of sequential image processing (ii-a to ii-g).

To improve the obtainable image quality, we undertook a series image processing of the raw images that were acquired by our Mλ-sMx-SPIM imaging system. Post-processing of microscope images was performed using MATLAB as well as ImageJ platforms. Figures S2-i(a-g) show the sequential images that were obtained after performing a series of processing. Figure S2, i.e., Fig. S2-i-a to Fig. S2-i-g, corresponds to the image of the fixed *Drosophila* sample for a particular section of the specimen. Whilst Fig. S2-ii-a to Fig. S2-ii-g present the corresponding maximum amplitude projection (MAP) images, after each image-processing step, that were obtained by projecting the corresponding 3D images into *xy*-plane or projecting along *z*-direction. Figure S2-i-a is the raw or unprocessed image while Fig. S2-ii-a is the corresponding MAP image obtained of the sequence of unprocessed stack of images. Figure S2-i-b shows the background subtracted image which is obtained by subtracting the dark current image from the unprocessed image by using MATLAB. Figure S2-ii-b shows the corresponding MAP image. In the next step, Contrast Limited Adaptive Histogram Equalization(CLAHE) was done using MATLAB. The processed image is given in Fig. S2-i-c and the MAP image is in Fig. S2-ii-c. Figure S2-i-d shows the median filtered image obtained (using MATLAB) and Fig. S2-ii-d gives the MAP image after median filtering. Further, processing was performed using ImageJ. Fig. S2-i-e and Fig. S2-ii-e give the background subtracted image and corresponding MAP image. Again, by using imageJ, brightness and contrast enhancement have been performed and the resulted images are shown in Fig. S2-i-f. The corresponding MAP image is shown in Fig. S2.ii-f. Finally, the remaining noise were removed by using denoise AI algorithm in Visual paradigm software (Fig. S2.i-g and Fig. S2.ii-g). The arrowhead in the magnified region (marked by yellow color) in Fig. S2-i-a and Fig. S2-i-g indicates the dorsal branch which was not observable in the unprocessed image but clearly visible

after all the steps of image processing. Similarly, the dorsal trunk in the marked region in Fig. S2-i(a-g) appears with better contrast and clarity following the sequential image processing steps. It is clearly observable that after undertaking CLAHE, the branch becomes more visible with better clarity (see Fig. S2-i-c). Still the image is associated with noises, which are successfully removed after performing median filtering (see Fig. S2-i-d). Later, the bright background is removed and contrast is further enhanced in the last two steps thereby giving image with better contrast and clarity. Even though the dorsal trunk and branches in the green marked region (Fig. S2-i-a) are visible before processing, the image (Fig. S2-i-f) gives better signal contrast, i.e., higher contrast of the targets with respect to the background, after the processing.

In the MAP images (Fig. S2-ii-(a-g)), the marked region (in red color) shows the dorsal branches. Here, we can clearly observe that the branches more distinct after performing CLAHE. But, in etha MAP images, the noises get accumulated. In order to remove the noises, we undertake background subtraction that enables to enhance the contrast and thus, recover the information with better quality. The red arrows (marked in Fig. S2-ii-e and Fig. S2-ii-g) indicate that the dorsal branches, that were lost in the process of maximum amplitude projection (MAP), are clearly recovered and visible after subtracting the background followed by enhancing brightness and contrast. By close observable of the image frames, it is clear that, after following our proposed post-processing, many features are visible in the MAP image that may be because of accumulation of from other frames or slices or accumulation of information from the entire volume. For better clarity, we present the zoomed-in perspectives (as insets) of the regions marked by the rectangle boxes that are indicated in Fig. S2-i-g and Fig. S2-i-g. In the figure, the arrowheads show the different branches. Since the MAP image gives the entire volume information in 2D, there will be a dimensional loss or wrapping but it provides a collective 3D render or information.

Supplementary 6: 360° Panoramic View of 3D Reconstructed Image

Our proposed Mλ-sMx-SPIM imaging system records the image of a 2D plane that is illuminated by a light sheet simultaneously on both detection arms. By translating the sample in a direction orthogonal to the light sheet, a sequence of images of different sections (*xy*-plane) of the whole sample can be recorded. A stack of the recorded images is then processed and used to reconstruct the 3D image by using Amira software. Video of 360° showing the panoramic view of the 3D images obtained at two different magnification (11.11× and 22.22×) are given in Supplementary Video S1. Supplementary Figure S3 gives representative images of the video showing the panoramic view.

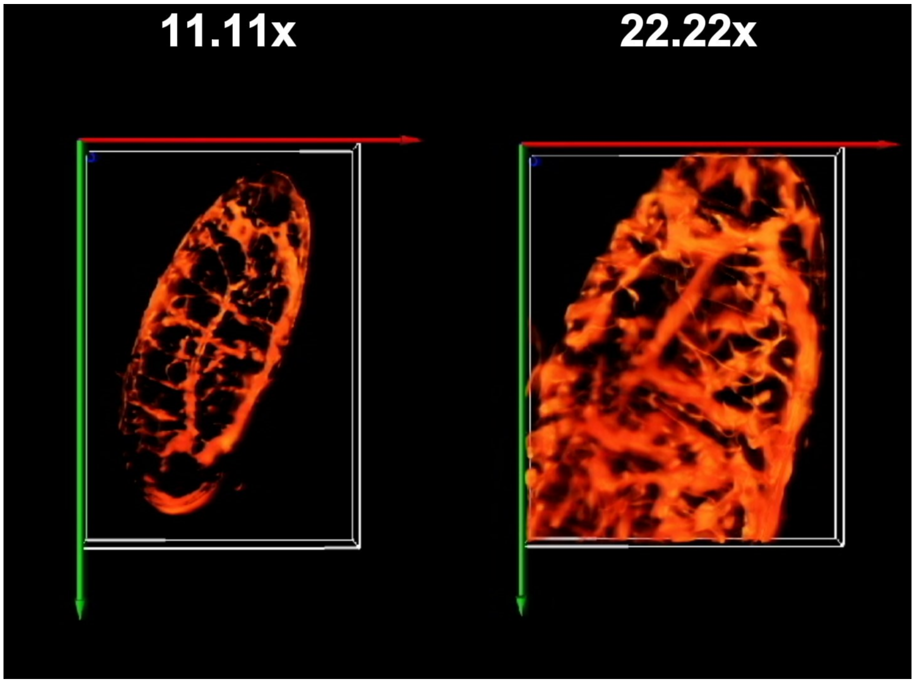

Figure S3: Thumbnail corresponding to Supplementary Video S1: 360° panoramic view of the 3D image at 11.11× and 22.22× magnifications.

Supplementary 7: Video Showing the Spatio-temporal Dynamics of Development of Tracheal System or Tracheal Branching in *Drosophila Melanogaster*

The live imaging of *Drosophila melanogaster* was carried out for an entire period of one hour at a time-interval of five-minute time. The video of the tracheal branching in both detection arms, for both the samples (Sample I and II) is given (2 frames/s). Sample I represents *Drosophila melanogaster* embryo progressing from developmental Stage 11-12 while Sample II represents embryonic developmental stages from late 13-15. For both the sample, we present two image frames of different magnification (11.11× and 22.22×) obtained at the same instant of time or simultaneously. See the movies Supplementary Video S2 (Sample I) and Supplementary Video S3 (Sample II) at two different magnifications (11.11× and 22.22×) obtained simultaneously or at the same instant of time. It is clear that with lower magnification we can observe wider field of view (but at lower resolution) while the higher magnification images give lower FOV (but at higher resolution and better clarity of minute contents). Shortly, with our proposed imaging system, one can observe and study of a given section of specimen at two separate FOVs that enables to observe simultaneously the whole structure of specimen as well as minute content in the specimen that remains as a technological challenge.

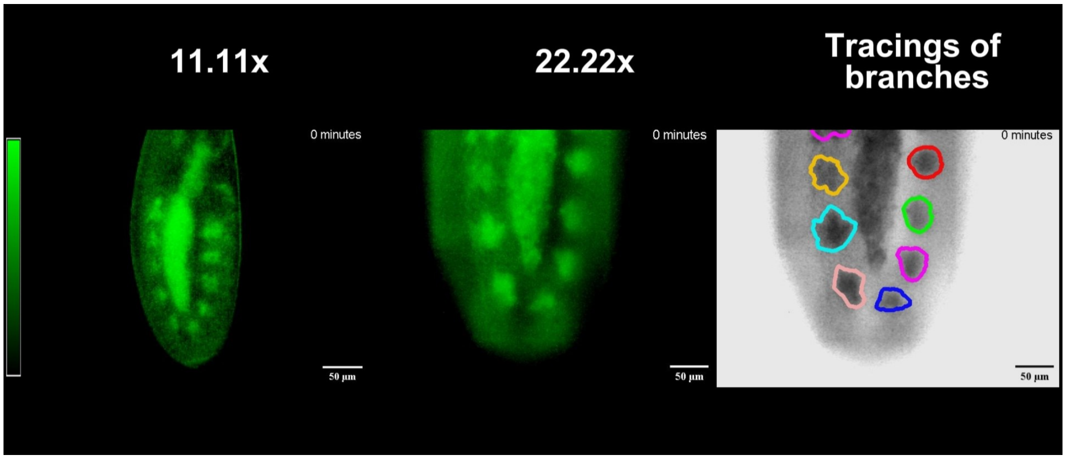

Figure S4-I: Thumbnail corresponding to Supplementary Video S2: Tracheal branching of *Drosophila* (Sample I) in the embryonic development Stage 11 to Stage 12 at 11.11× and 22.22× magnifications.

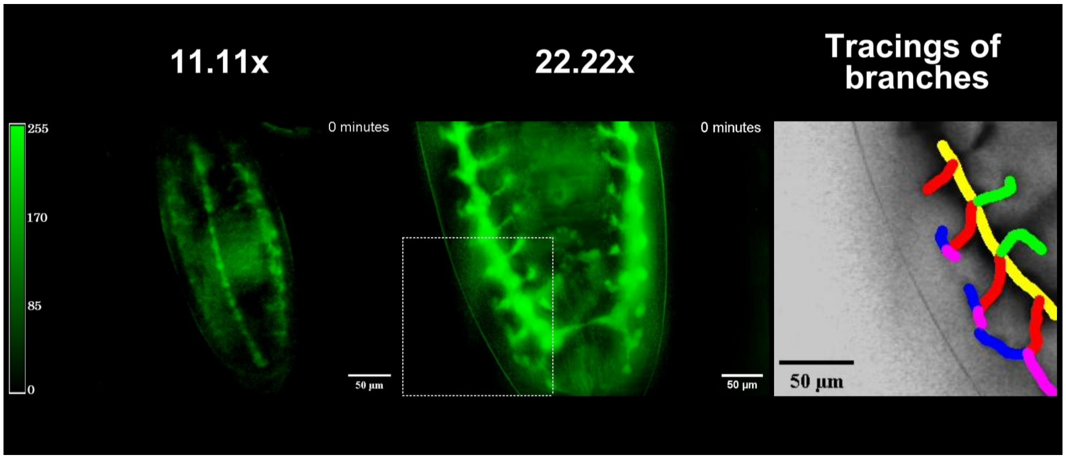

Figure S4-II: Thumbnail corresponding to Supplementary Video S3: Tracheal branching of *Drosophila* (Sample II) in the embryonic development late Stage 13 to Stage 15 at 11.11× and 22.22× magnification.

Supplementary 8: Quantitative Characterization of the Development and the Growth of Tracheal Branches in *Drosophila Melanogaster*

section gives the quantitative characterization of the development or growth of various tracheal branches in *Drosophila melanogaster*. In true sense, we measured the branch-lengths of the individual tracheal branches and the areas of tracheal pits

using a combined software of NeuronJ and ImageJ. Figure S5-I depicts the bar-plot showing the area of tracheal tissue with respect to time (t), i.e., temporal growth of tissue area, for different tracheal pits (as they are indicated by marking in Fig. 5 in the main article). The figure demonstrates that the tracheal tissue areas are significantly increased in the duration of 60 minutes (say, from $t0$ to $t0+60min.$). Figure S5-II presents the changes in the branch lengths of all the tracheal branches that include TC, DT, aLLT, and pLLT (see Fig. 2 in main article and Supplementary Fig. S5-II). This may be noted that, in Fig. 6 (main article), we present the changes in branch lengths for selective tracheal branch (because of limited space). For this study of changes in tracheal branch length, we employed Sample II and we employed $22.22\times$ magnification. The development and growth of tracheal branches were recorded for an entire duration of 95 minutes at which the tracheal branches lengths are found to be saturated, i.e., growth of tracheal branches are matured (see Fig. 5 (main article)). For calculation of tracheal pit area and branch length, we employed NeuronJ and ImageJ combinedly. In both of the studies, we can see an increase in the area of the tracheal tissues or tracheal branches, i.e., tracheal growth.

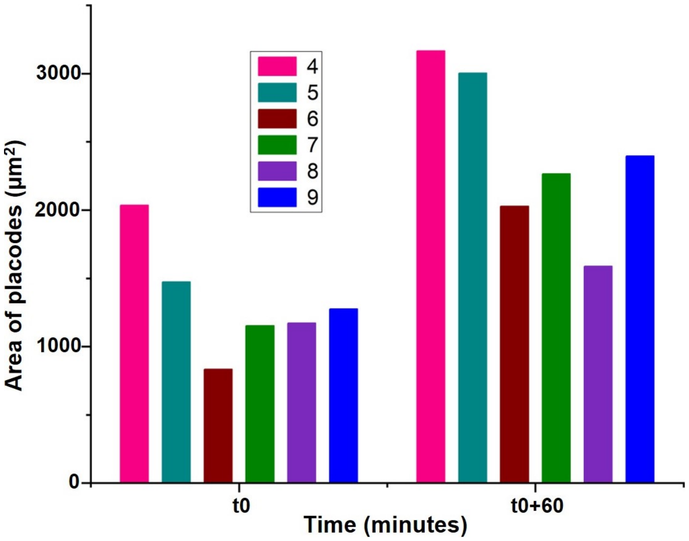

Figure S5-I: Comparison of area of the tracheal pits in Sample I at the beginning and after one-hour of imaging. The labeling of tracheal pits are mentioned in Fig. 8.I-f and Fig. 8.I-j.

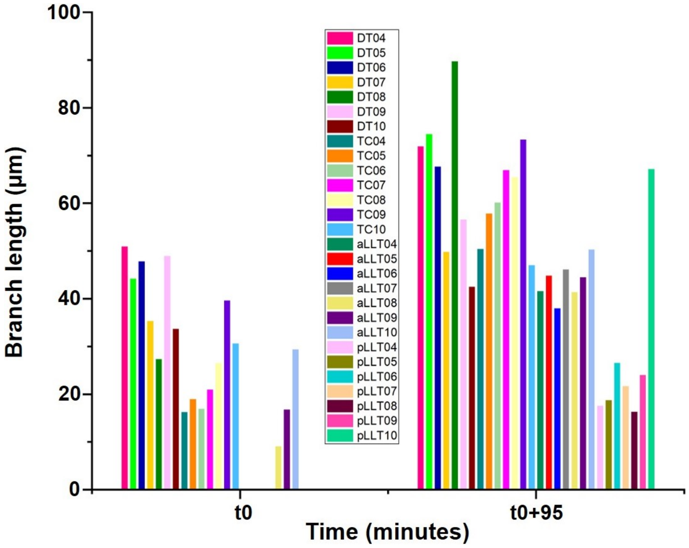

Figure S5-II:Comparison of branch lengths of all tracheal branches of Drosophila melanogaster (Sample II) at the beginning and after the interval of 95 minutes, i.e., at $t0$ and $t0+95$.

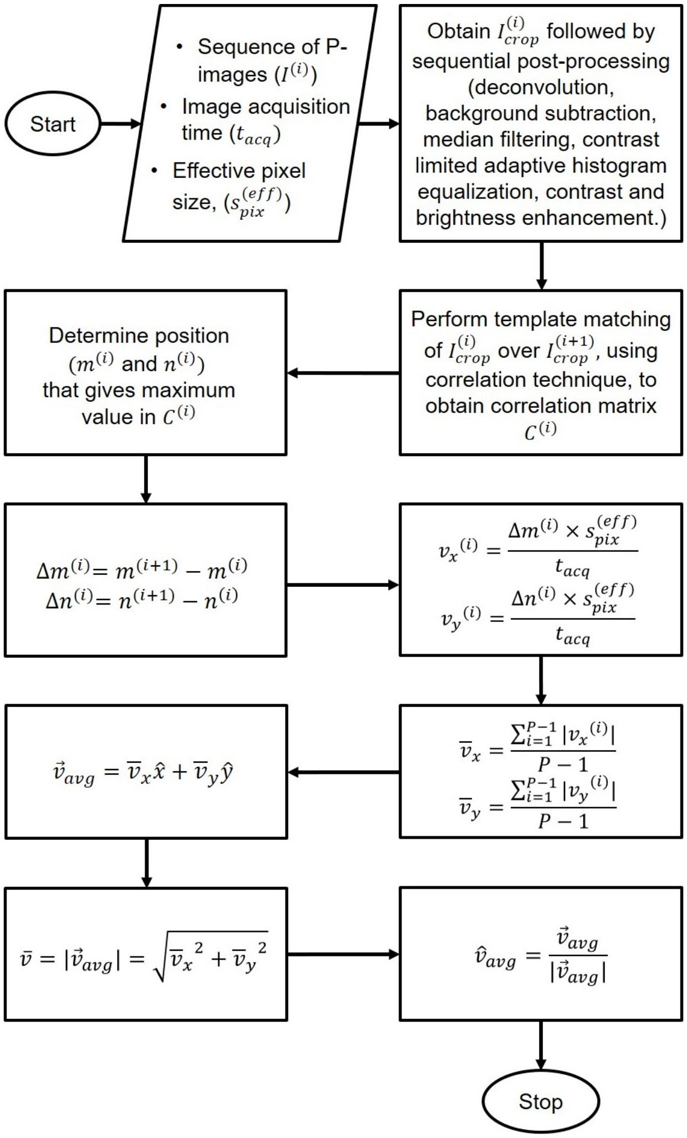

Figure S6: Flow chart or algorithm of the custom-made (MATLAB) program to estimate the velocity (both magnitude and direction) of mitochondria.

Supplementary 9: Algorithm with flowchart to find the speed of mitochondria

Figure S6 depicts a flow-chart representative of the algorithm that we employed for estimating the velocity ($\vec{v}$) of individual mitochondria (both magnitude ($|\vec{v}|$) and direction ($\hat{v}$)). In the algorithm, as the first step, we read a sequence of image frames ($I^{(i)}$, $i = 1,2,3,....,P$ (say)) that we captured experimentally with our Mλ-sMx-SPIM imaging system. Image acquisition time (t_{acq}) (or image frame rate) and pixel size of the camera (s_{pix}) were taken as inputs. Subsequently, the images were cropped over a particular region of interest (ROI) − more specifically, over particular mitochondria of interest − and this gives $I_{crop}^{(i)}$ ($i = 1,2,3,....,P$). For each two consecutive cropped images ($I_{crop}^{(i)}$ and $I_{crop}^{(i+1)}$), we estimated a correlation matrix ($C^{(i)}$, $i = 1,2,3,....,P$) where each matrix element (at $[m,n]$) represents the estimated value of the correlation of the two consecutive image frames ($I_{crop}^{(i)}$ and $I_{crop}^{(i+1)}$) while the image ($I^{(i+1)}$) is scanned over pixel-by-pixel ($[m,n]$) by the template or reference image ($I^{(i)}$). Then, corresponding to the maximum value of correlation in the correlation matrix ($C^{(i)}$), we determined the distance through which the mitochondria move (in the $(i+1)^{\text{th}}$ image frame), more specifically, along x-direction (Δx) and y-direction (Δy) are separately given by $\Delta m^{(i)} \times s_{pix}^{(eff)}$ and $\Delta n^{(i)} \times s_{pix}^{(eff)}$. Here, $\Delta m^{(i)} = m^{(i+1)} - m^{(i)}$ and $\Delta n^{(i)} = n^{(i+1)} - n^{(i)}$ are the number of pixels through which the image template is shifted (along x-direction and y-direction) corresponding to the maximum correlation value. $s_{pix}^{(eff)}$ is the effective pixel-size. With the given image acquisition time (t_{acq}) which corresponds to

the time duration for mitochondria to traverse in the two consecutive (image) frames, we can determine the velocity components (v_x and v_y) and we took an average over the entire number of image frames ($P-1$) to give $\bar{v}_x$ and $\bar{v}_y$. From this, we can obtain the velocity vector ($\vec{v}_{avg} = \bar{v}_x\hat{x} + \bar{v}_y\hat{y}$). Finally, we can achieve velocity magnitude ($|\vec{v}_{avg}| = \sqrt{\bar{v}_x^2 + \bar{v}_y^2}$) and its unit vector $\hat{v}_{avg} = \frac{\vec{v}_{avg}}{|\vec{v}_{avg}|}$.

The effective pixel size ($s_{pix}^{(eff)}$) is calculated from the magnification (M) and pixel size (s_{pix}) of the camera that is given by:

$$s_{pix}^{(M)} = \frac{s_{pix}}{M}, \tag{1}$$

where M is the optical magnification power of the detection arm in which the camera is fitted as an optical sensor array. In our studies, the acquired images are re-sized to double using linear interpolation. Thus, the effective pixel size ($s_{pix}^{(eff)}$) of the pixel is given by:

$$s_{pix}^{(eff)} = \frac{s_{pix}^{(M)}}{2}. \tag{2}$$

For our case with camera (BFS-U3-27S5M-C) of pixel size ($s_{pix} = 4.5\ \mu m$) and magnification ($M = 44.4$), $s_{pix}^{(M)}$ and $s_{pix}^{(eff)}$ are estimated to be 0.10 μm and 0.05 μm respectively.

Supplementary 10: Video Showing the Spatio-temporal Dynamics of Organelles (Mitochondria)

This section presents study on spatio-temporal dynamics of mitochondria (in HeLa cell) where we employed 44.44× magnification for capturing the time-resolved sequential images or video. Supplementary Video S4 give a typical video showing the spatio-temporal dynamics of mitochondria in the 44.44× magnification and zoomed-in views at four different or separate regions. The time-resolved sequential images were recorded at the frame rate of 17 frames/s, i.e., the image acquisition time (t_{acq}) is estimated to be $\sim$ 0.44 s. Figure S7 gives a representative image being randomly selected from the video or sequential images. In the zoomed-in views, one can clearly identify individual mitochondria. These sequential images were employed quantitative study of spatio-temporal dynamics — more specifically, velocity ($\vec{v}$) both magnitude ($|\vec{v}|$) and direction ($\hat{v}$) — the details of which are presented in Supplementary 9. The sequential images were post-processed and individual and/or clusters of mitochondria were tracked using our custom-made (MATLAB) program. The tracked mitochondria were marked using boxes of different colors.

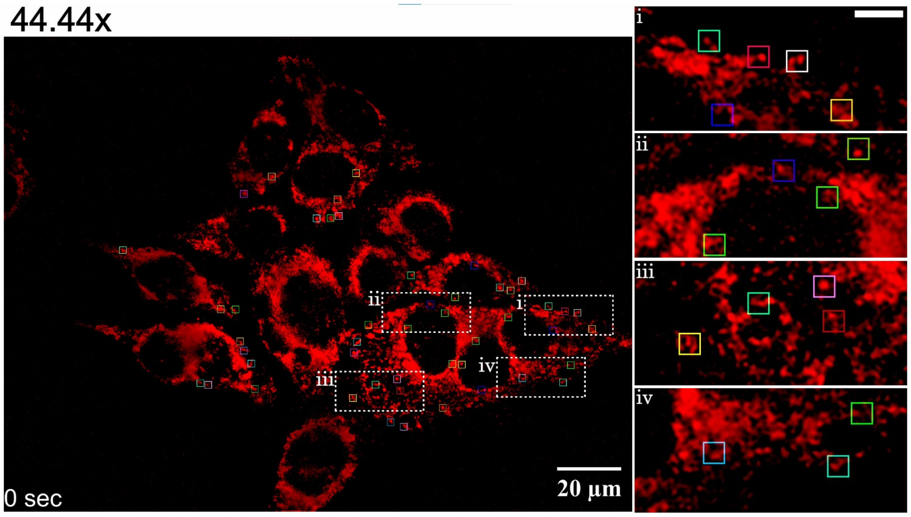

Figure S7: Thumbnail corresponding to Supplementary Video S4: Tracking of mitochondria by using a custom-made program. In insets, zoomed-in views of the four different or separate regions (as they are indicated by marked boxes in the figure) are depicted.

Supplementary 11: Tracing and Labeling of Spatio-temporal Dynamics of Organelles (Mitochondria)

Figure S8 depicts labelling of mitochondria of interest (in HeLa cell) that were selected for the quantitative study of spatio-temporal dynamics (more specifically, velocity ($\vec{v}$)). The mitochondria were selected different and diversified regions, namely,

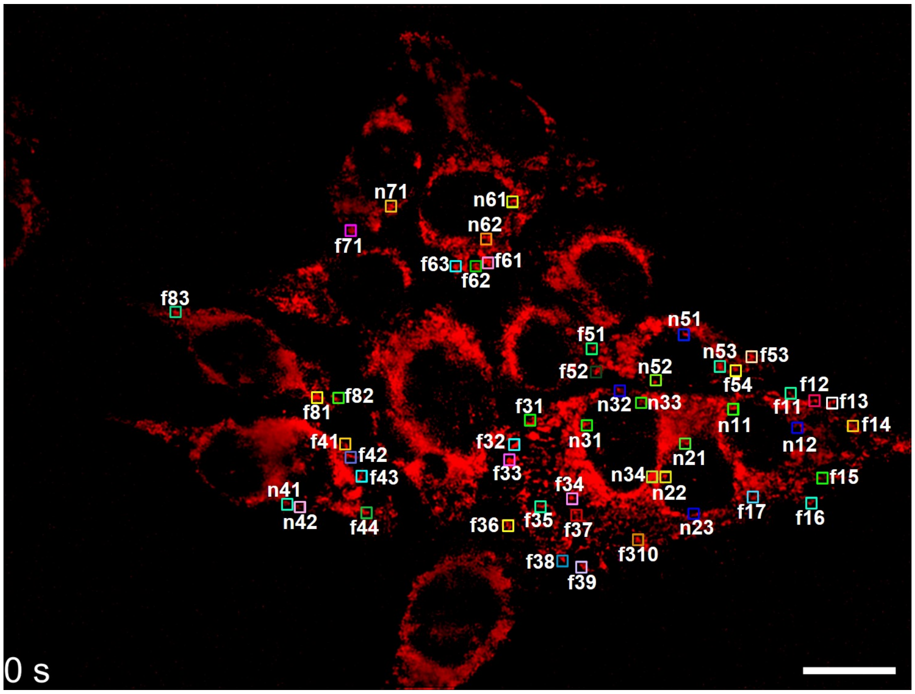

Figure S8: Labeling of cells and organelles (mitochondria) of cells. Scale bar: 20 μm.

close to nucleus and periphery of the cell (or away from nucleus). We aso chose mitochondria from three classes of cells: (i) isolated cells (not interacting), (ii) cluster (highly interacting) cells, and (iii) moderately interacting cells. In true sense, this is for the requirement to conduct correlation studies of the spatio-temporal dynamics (speed) of mitochondria in different types of cells and at different distances from the nucleus. Each mitochondrion or small cluster of mitochondria − for which speed is calculated − is marked and labeled separately for different cells (see Supplementary Fig. S8). In the labelling, the first letter ‘n’ or ‘f’ represents whether the mitochondrion is near or far from the nucleus. The second letter is a number that represents the label of the cell while the third letter represents the label of mitochondrion in that cell. For example, the ‘n12’ label represents the second mitochondria of the first cell near the nucleus.

References

1. Hayashi, S. & Kondo, T. Development and function of the drosophila tracheal system. *Genetics* **209**, 367–380 (2018).
2. Samakovlis, C. *et al.* Development of the drosophila tracheal system occurs by a series of morphologically distinct but genetically coupled branching events. *Development* **122**, 1395–1407 (1996).
3. Ritter, J. G., Veith, R., Veenendaal, A., Siebrasse, J. P. & Kubitscheck, U. Light sheet microscopy for single molecule tracking in living tissue. *PloS one* **5**, e11639 (2010).
4. Tokunaga, M., Imamoto, N. & Sakata-Sogawa, K. Highly inclined thin illumination enables clear single-molecule imaging in cells. *Nat. methods* **5**, 159–161 (2008).
5. Rinsa, S., Manoj, B. & Singh, M. S. Investigation of nuclear structural variations under chronic stress conditions in allium cepa using lsfm. In *Imaging, Manipulation, and Analysis of Biomolecules, Cells, and Tissues XIX*, vol. 11647, 28–34 (SPIE, 2021).
6. Regmi, R., Mohan, K. & Mondal, P. P. Light sheet based imaging flow cytometry on a microfluidic platform. *Microsc. research technique* **76**, 1101–1107 (2013).
7. Regmi, R., Mohan, K. & Mondal, P. P. High resolution light-sheet based high-throughput imaging cytometry system enables visualization of intra-cellular organelles. *AIP Adv.* **4** (2014).
8. Krzic, U., Gunther, S., Saunders, T. E., Streichan, S. J. & Hufnagel, L. Multiview light-sheet microscope for rapid in toto imaging. *Nat. methods* **9**, 730–733 (2012).
9. Lander, A. D. Pattern, growth, and control. *Cell* **144**, 955–969 (2011).

10. Mondal, P. & Diaspro, A. *Fundamentals of Fluorescence Microscopy: Exploring Life with Light* (2013).
11. Saleih, B. & Teich, M. Fundamental of photonics (1991).
12. Jenkins, F. & White, H. Fundamental of optics (1976).
13. Ghatak, A. Optics (2009).
